# From manganese mineral evolution history to atmospheric oxygen reconstruction

**DOI:** 10.1093/nsr/nwag230

**Published:** 2026-04-17

**Authors:** Yan Li, Ziyi Zhuang, Xinran Xu, Rongzhang Yin, Yanzhang Li, Yadong Wang, Chunjiang Li, Yong Lai, Yanan Zhang, Huan Ye, Zhaoyang Hu, Anhuai Lu, Robert M Hazen, Xiangzhi Bai

**Affiliations:** SKLab-DeepMinE, MOEKLab-OBCE, School of Earth and Space Sciences, Peking University, Beijing 100871, China; Beijing Key Laboratory of Mineral Environmental Function, School of Earth and Space Sciences, Peking University, Beijing 100871, China; Beijing Key Laboratory of Mineral Environmental Function, School of Earth and Space Sciences, Peking University, Beijing 100871, China; Image Processing Center, Beihang University, Beijing 102206, China; SKLab-DeepMinE, MOEKLab-OBCE, School of Earth and Space Sciences, Peking University, Beijing 100871, China; Beijing Key Laboratory of Mineral Environmental Function, School of Earth and Space Sciences, Peking University, Beijing 100871, China; SKLab-DeepMinE, MOEKLab-OBCE, School of Earth and Space Sciences, Peking University, Beijing 100871, China; Beijing Key Laboratory of Mineral Environmental Function, School of Earth and Space Sciences, Peking University, Beijing 100871, China; Image Processing Center, Beihang University, Beijing 102206, China; SKLab-DeepMinE, MOEKLab-OBCE, School of Earth and Space Sciences, Peking University, Beijing 100871, China; Beijing Key Laboratory of Mineral Environmental Function, School of Earth and Space Sciences, Peking University, Beijing 100871, China; SKLab-DeepMinE, MOEKLab-OBCE, School of Earth and Space Sciences, Peking University, Beijing 100871, China; Image Processing Center, Beihang University, Beijing 102206, China; SKLab-DeepMinE, MOEKLab-OBCE, School of Earth and Space Sciences, Peking University, Beijing 100871, China; Beijing Key Laboratory of Mineral Environmental Function, School of Earth and Space Sciences, Peking University, Beijing 100871, China; SKLab-DeepMinE, MOEKLab-OBCE, School of Earth and Space Sciences, Peking University, Beijing 100871, China; Beijing Key Laboratory of Mineral Environmental Function, School of Earth and Space Sciences, Peking University, Beijing 100871, China; Beijing Key Laboratory of Mineral Environmental Function, School of Earth and Space Sciences, Peking University, Beijing 100871, China; Earth and Planets Laboratory, Carnegie Institution for Science, Washington, DC 20015, USA; Image Processing Center, Beihang University, Beijing 102206, China; State Key Laboratory of Virtual Reality Technology and Systems, Beihang University, Beijing 100191, China; Advanced Innovation Center for Biomedical Engineering, Beihang University, Beijing 100083, China

**Keywords:** mineral informatics, Mn mineral dataset, atmospheric oxygenation history, Mn crystal chemistry, deep learning

## Abstract

The evolutionary record of redox-sensitive manganese (Mn) minerals encodes critical information about Earth’s oxygenation history. By building a global Mn mineral dataset (144 200 entries across 25 feature dimensions), we developed a URD (Unequal-size feature matrix, Recoupling relationship, and Disaccord labels) deep-learning model to reconstruct continuous atmospheric oxygen level (*p*O_2_) changes over 4.0 billion years. Our results provide robust mineralogical evidence linking the timing and tempo of oxygenation to planetary-scale tectonics and biosphere evolution. Specially, the reconstruction reveals two distinct oxygenation modes: a protracted and gradual increase during the Paleoproterozoic–Mesoproterozoic, reflected in the moderately progressive evolution of Mn mineral assemblages; and a more rapid rise preceding and following the Neoproterozoic, coincided with supercontinent breakup and convergence, respectively—a pattern potentially driven by tectonic modulation of Mn supply and demand. This study introduces a mineral-informatic framework for decoding complex, high-dimensional mineral records, offering a transformative approach for systematically interrogating Earth’s long-term evolution.

## Teaser

This work presents an Unequal-size feature matrix, Recoupling relationship, and Disaccord labels deep-learning model designed for mineralogical big data. The model decodes the coevolutionary relationships between *p*O2 and the phylogenetic histories of manganese minerals and reconstructs Earth’s atmospheric oxygenation history over nearly 4.0 Gyr.

## INTRODUCTION

Understanding the evolution of Earth’s atmospheric oxygenation over the past 4.0 billion years and utilizing the existing Earth records to infer changes in O_2_ concentration are fundamental challenges for revealing the coevolution of both Earth’s interior and near-surface environments [1,2]. Abundant prior researches dedicated to estimating historic atmospheric O_2_ levels (*p*O_2_) have largely relied on geochemical models, including redox-sensitive proxies and isotope indicators [3–5]. However, the history of Earth’s atmospheric oxygenation remains elusive due to the lack of continuous and complete spatiotemporal geological records of these typical proxies. Although a continuous atmospheric O_2_ curve has been reconstructed from big data of igneous rock records [6], its evolution pattern may not be fully consistent with *p*O_2_, given that igneous rocks mainly reflect the redox state of deep Earth. Additionally, fragmented reconstructions of *p*O_2_ by these modeling approaches often yield disagreed results due to the different sensitivities of those proxies to changes in O_2_ fugacity [7–12]. While continuous ice core records offer quantifiable accuracy in directly detecting *p*O_2_, they are limited in long-term estimations [13,14]. Therefore, there is an urgent need for a unified proxy that combines pan-geological temporal coverage with high redox sensitivity.

Manganese (Mn) minerals emerge as an ideal candidate for *p*O_2_ reconstruction due to their unique geochemical properties. As Earth’s second most abundant transition metal with three valence states in crustal environments, Mn exhibits exceptional sensitivity to environmental redox states. Both the distribution range and total categories of Mn minerals far exceed those containing most other redox-sensitive metals such as Ce (168 mineral species) and Cr (109 mineral species) (statistics from RRUFF database). The currently known 634 distinct Mn mineral species (https://rruff.info/ima; accessed December 2021) are distributed at all crustal depths and latitudes [15]. Their compositional and structural diversity reflects a broad range of O_2_ fugacity, from tephroite (Mn^II^_2_[SiO_4_]) and pyroxmangite (Mn^II^[SiO_3_]) at low O_2_ fugacity to pyrolusite (Mn^IV^O_2_) at the highest O_2_ fugacity [16]. Critically, Mn minerals provide more direct *p*O_2_ records than conventional redox-sensitive metals and their isotope proxies, like Mo, Cr, or Ce. Because such redox-sensitive proxies are directly oxidized by Mn oxides rather than oxygen, while the reducing forms of Mn subsequently consume oxygen for regeneration [17–20], which suggests that Mn minerals have a more direct interaction with *p*O_2_. These prior studies indicate that changes in global Mn mineral suites directly reflect *p*O_2_ variations.

However, decoding the oxygenation record preserved in Mn minerals requires addressing several fundamental complexities. First, although the mineralization frequency and overall oxidation states of supergene Mn minerals have been revealed to be heavily influenced by the oxygenation of atmosphere and oceans [21], the geochemical cycle of Mn in subsurface regions is also controlled by *p*O_2_ variations through plate tectonic-mediated energy–mass exchange [22,23]. This coupled surface-deep redox dynamics determines that the mineral–*p*O_2_ coevolving relationship is inherently nonlinear. Second, Mn minerals exhibit multidimensional responses to *p*O_2_ changes. Their valence states, chemical compositions, and crystal structures, collectively (yet differentially) respond to *p*O_2_ fluctuations. This constitutes a challenging many-to-one relationship between mineralogical features and oxygen levels. Moreover, the same mineral species can form under different *p*O_2_ conditions, while different minerals may form under similar *p*O_2_. This creates complex time-transgressive relationships.

Methodological challenges further arise when employing large mineralogical databases to unravel these relationships. The coevolution of Mn minerals and *p*O_2_ is characterized by unequal feature length of Mn mineral data, disaccord *p*O_2_ labels, and recoupling relationships among contemporaneous Mn minerals, specifically, the varying Mn mineral species corresponding to different ages, leading to unequal feature matrix sizes of Mn mineral samples at different ages. The problem of disaccord labels, stems from the wide range of geologic ages of Mn mineral occurrences, resulting in inexplicit correspondence between Mn minerals and *p*O_2_. In addition, the exact relationship between each Mn mineral and *p*O_2_ is also ambiguous, especially the same Mn mineral may appear at different ages corresponding to different *p*O_2_ or different Mn minerals may appear at the same age corresponding to the same *p*O_2_. This causes the complex recoupling relationship between the Mn mineral species that existed at the same or different ages.

In this work, we present a deep-learning strategy to elucidate this intricate coevolution relationship and reconstruct the continuous variations of *p*O_2_ over the past 4.0 Gyr. Our approach establishes quantitative relationships between the integrated multidimensional characteristics of global Mn mineral assemblages and *p*O_2_ fluctuations, yielding oxygenation reconstructions with superior objectivity and temporal resolution. Moreover, uncovering the correlations between *p*O_2_ and distinct sets of Mn minerals across different geological epochs also provides new insight into the complex and dynamic coevolution mechanisms of Mn minerals and Earth’s oxygenation events. This research also showcases the robust capabilities of mineral informatics by introducing artificial intelligence tools into geoscience data mining and interpretation.

## RESULTS AND DISCUSSION

### Deep-time coevolution of Mn minerals and atmospheric O_2_

After Hummer *et al.* [21], who counted the number of Mn mineral-locality pairs in different Mn valence states, we screened the original data (more details in Methods section) and further built a dataset including 476 Mn mineral species with 144 200 characteristic data points of 5768 Mn mineral samples collected from both the RRUFF (https://rruff.info/evolution) and Mindat (https://mindat.org/) (up to December 2021). All dated Mn minerals in this dataset include not only low-grade Mn as dispersed Mn minerals, but also high-grade Mn ores (i.e. economic Mn deposits). Each Mn mineral was represented by 25-dimensional features (Table S1), including geologic age (two-dimensional features), chemical composition (three-dimensional features), key elements (seven-dimensional features), Mn valence states (three-dimensional features), structural symmetry (nine-dimensional features), and chemical classification (one-dimensional feature). In addition, we created a *p*O_2_ dataset containing 552 data points from relevant literatures, including 526 data points from ice core records and 26 data points reconstructed from geochemical proxies and models (Table S2).

**Figure 1.**
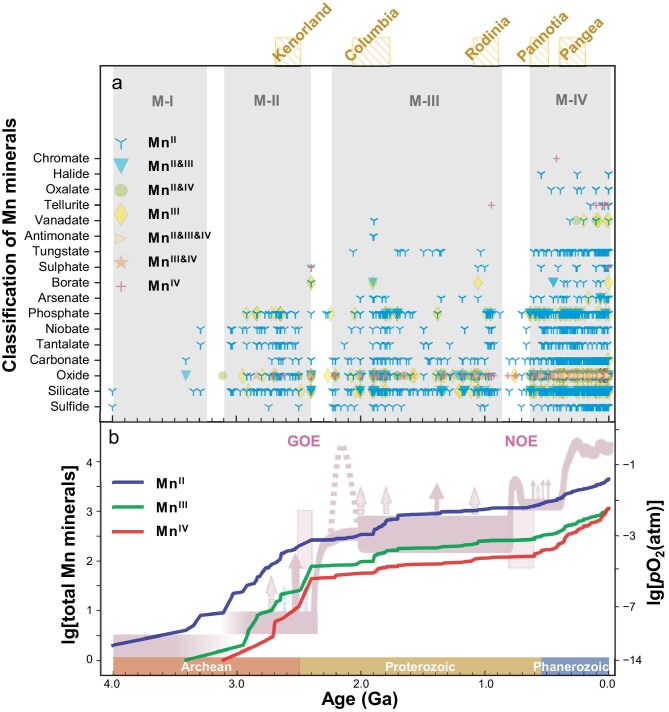
Deep-time coevolution of Mn minerals and *p*O_2_ in Earth. (a) Scatter diagram of Mn minerals evolution over 4.0 Ga (labeled as different classification and Mn valence states). Gray bands labeled by Roman numerals with prefix M represent four mineral evolution stages. (b) Broken line diagram showing the changing trends of Mn^II^, Mn^III^, and Mn^IV^ mineral counts through time. Shown in pink are current views on *p*O_2_ and the approximate timing of the GOE and the NOE, taken from Lyons *et al.* [2]. One mineral count is defined as one occurrence of sample-locality pair. All ages of minerals plotted here are their maximum ages.

As shown in Fig. 1, the diversity of Mn minerals increased through time, and the statistical counts in three different valence states showed a consistent trend, where their increasing rates roughly agree with the current view on the changing rhythm of *p*O_2_ [2]. A great majority of Mn minerals (468 species) arose after the Great Oxidation Event (GOE), and >70% of Mn minerals were formed in sedimentary and supergene processes (Table S3). According to the observed changes in mineralization frequency, continuity, and variety of different subdivided mineral categories, the evolution of Mn minerals was divided into four stages (Fig. 1a and Table S3).

In Stage M-I (4.0 to 3.2 Ga), only limited types of Mn^II^-bearing minerals mainly as magmatic hydrothermal silicates were observed. Some Mn minerals like Mn^III^ (oxide), C^IV^ (carbonate), Nb^III^ (niobate), and Ta^V^ (tantalate) in relatively oxidized states were also sporadically recorded. It should be noted that the mineral record for Stage M-I is too sparse and limited to draw definitive conclusions.

Stage M-II occurred between 3.1 and 2.4 Ga, witnessing a rapid increase in the diversities of Mn^II^, Mn^III^, and Mn^IV^ minerals. This period also marked the emergence of various oxysalts, including phosphate, arsenate, borate, and sulphate, whose successive appearance indicates a substantial enhancement in Mn mobility during this stage. This mineral diversification was correlated to changes in the near-surface geochemical environment, such as increasing free O_2_ and/or biological activities. The most significant change was the appearance of Mn^III^ and Mn^IV^ oxides, indicating at least local oxygenation occurred in the near-surface environments.

Stage M-III (2.2 to 0.9 Ga) shows a mineralization tempo similar to that of the second stage, but with an obvious increase in both categories and deposition frequency of Mn oxysalt minerals of low-temperature genesis. These changes suggest an increasing oxygen level and enhanced weathering, which resulted in further accumulation of mobile Mn and led to the precipitation of new minerals. Later, most of the low-temperature Mn oxysalts display sedimentary discontinuity during the Neoproterozoic interval 0.8 to 0.6 Ga, until their recovery in Stage M-IV. The fourth stage was identified from the full bloom of the majority of Mn mineral species in the past 0.65 Ga (Table S3). Due to the significant increase in *p*O_2_, which approached the present level, extensive oxidative weathering facilitated the enrichment of redox-sensitive elements such as Sn, V, As, B, W, Sb, Te, and Cr, resulting in the formation of various Mn oxysalts with relatively higher solubility than previous Mn minerals. Besides, the rising atmospheric O_2_ levels led to increased concentrations of oxyacid anions (such as SO_4_^2−^, C_2_O_4_^2−^, etc.) in the aqueous environment, which also promoted the formation of highly soluble oxysalt minerals. As shown in Fig. S1, the appearance of high‑solubility classifications (chlorides/sulfates/oxalates) indicate an increase in dissolved Mn flux.

The evolution records of Mn minerals reveal a systematic increase in both their diversity and deposition frequency over billions of years of Earth’s history, particularly among oxides and low-temperature oxysalts (Fig. 1 and Table S3). In addition, Mn minerals with greater solubility than those in previous stages gradually emerged, representing the cumulative enrichment of Mn in the supergene environment where atmosphere, hydrosphere, lithosphere, pedosphere, and biosphere closely interact. Furthermore, the explored reserves and age data for all economic Mn deposits since 3.5 Ga were collected and plotted in Fig. S2. The economic mineralization of large-scale Mn deposits through time exactly occurred at the time with dramatically increasing *p*O_2_, which once again proves the close relationship between Mn mineralization and atmospheric O_2_ [24,25]. Notably, when *p*O_2_ was further increased, such as at the Neoproterozoic Oxygenation Event (NOE), the subsequent further increases in the diversity, deposition frequency, and scale of Mn minerals are also the corresponding feedbacks to the changes in *p*O_2_.

Although the oxygenation process played strong roles in the diversification of Mn minerals, determining the causality between the variations in *p*O_2_ and Mn minerals is complex. For example, the evolution of Mn mineralization is complicated by the universal biological effects on Mn cycling. Organisms exhibit metabolic versatility in the face of fluctuating O_2_ availability, releasing diversified metabolite products to the environments [26]. Environmental changes like this commonly led to new collections of atoms forming new minerals. Therefore, variations in Mn minerals reflect (bio)geochemical responses to fluctuating O_2_ levels. These responses include a series of electron transport chain reactions that eventually link to atmospheric O_2_ as the terminal electron acceptor.

### O_2_ level reconstruction by deep-learning model: URD

**Figure 2.**
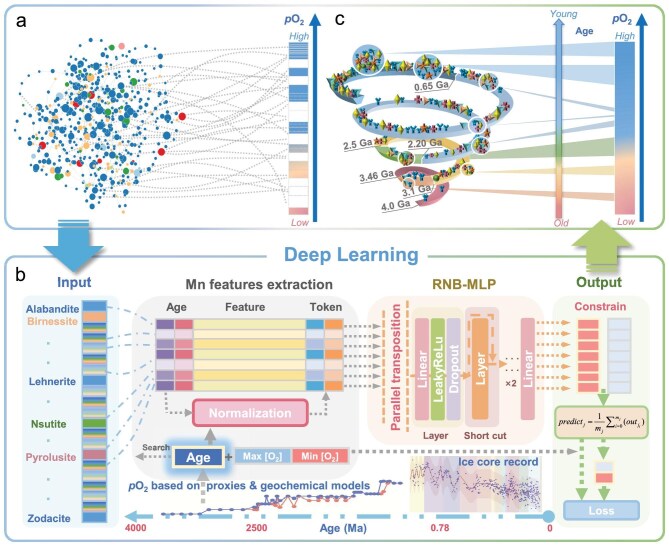
Cartoon representing the deep-learning method (URD network) to establish the time series mapping relationship between a specific Mn mineral set with *p*O_2_. (a) The Mn mineral data established in the text (on the left: one circle represents one Mn mineral, different color represents different Mn valence states, and the circle size represents the number of samples) and the discrete oxygen data collected from literatures (the colored vertical bars on the right). The dashed curve represents the correspondence between each Mn mineral and *p*O_2_ of its formation environment. (b) Schematic diagram of the developed URD network to build up the evolution of Earth’s *p*O_2_. A Mn feature extraction block introduced time-varying characteristics of Mn minerals through the normalization layer. RNB-MLP block converted parallel batch processing on features into a length-adaptive serial processing by a parallel transposition layer. Logarithmic loss function was constructed to handle the exponentially-scaled changes in *p*O_2_. (c) There exists a significant correlation between the evolution of Mn minerals (the small symbols represent Mn minerals) and *p*O_2_. Application of the URD model revealed a one-to-one mapping relationship between *p*O_2_ and a specific Mn mineral set (shown in different circles) at any geologic age.

In essence, the thermodynamic properties of minerals are determined by all of their crystal chemical characteristics. Therefore, the coevolution information of *p*O_2_ and Mn minerals should be reflected in the crystal chemical characteristics of all the constituent minerals at the same period, not only the valence information of a certain mineral or element. Considering the high complexity in data matching of Mn minerals–*p*O_2_ datasets (Fig. 2a), we constructed a URD (Unequal-size feature matrix, Recoupling relationship, and Disaccord labels) deep-learning network (details in subsection *URD model* of *Methods* section) to analyse the coevolution regulation of Mn minerals and *p*O_2_ versus time (Fig. 2c). The network input was *m_t_* × 25-dimensional features of Mn mineral samples (*m_t_*) at age *t*, and the output was predicted *p*O_2_ at age *t*. The Mn feature extraction block introduced time-varying characteristics of Mn minerals through a normalization layer and constructed a ‘time token’ to mark the relative stage in the life cycles of current Mn mineral samples, so that the features strictly corresponding to *m_t_* at age *t* were generated to drive RNB-MLP (Residual Multi-Layer Perception without Batch Normalization). This high-dimensional input that combined different properties of Mn minerals and the special treatment of age ranges, together with *p*O_2_ predictions in the form of ranges, allowed URD model to better describe this nonlinear coupling relationship with time.

As a result, it is capable of learning the relationship between mineral thermodynamic information and crystal chemistry characteristics, making it different from conventional geochemical proxies. RNB-MLP converted parallel batch processing on features into a length-adaptive processing by a parallel transposition layer, which allowed us to solve the difficult forward propagation problem caused by the uneven number of Mn mineral samples at each geologic age *t*. The logarithmic loss function constructed an exponential metric of the prediction error to alleviate the negative impact of the long-tailed data distribution caused by exponential *p*O_2_ change between 10^−13^ and 1 PAL (present atmospheric level).

To assess the performance of the URD model, we conducted a comprehensive evaluation. The model not only successfully predicted continuous *p*O_2_ variations across the entire 4.0 Gyr interval, but also exhibited high prediction accuracy (Fig. S3), strong stability, and significantly improved temporal resolution compared to previous methods. The Mean Square Error (MSE) and Mean Absolute Percentage Error (MAPE) for the predictions on verification data were very small: 0.071 and 0.073 (upper and lower bounds of *p*O_2_) for MSE, and 0.042 and 0.045 for MAPE, with an *R*-squared (*R*^2^) as high as 0.990 and 0.991. To further validate the generalization ability of the URD model, 10-fold cross-validation experiments were performed (Table S4), with average results confirming robustness (MAPE < 0.2, MSE < 0.2, and *R*^2^ > 0.96).

We also compared the URD model against existing methods, including the Pad Zero [27], Identification [28], and Mean Feature [29] methods. The URD model outperformed all alternatives (see Supplementary material for details, Table S5). Furthermore, to evaluate cross-proxy generality, we tested the model using an independent molybdenum (Mo) mineral dataset (1366 samples from RRUFF) as external validation, which is smaller and more temporally uneven than the Mn dataset (Fig. S4). Despite these differences, the reconstructed *p*O_2_ curve for 0−1.0 Ga shows remarkable consistency with Mn-based results (Fig. S5), and performance metrics (Table S6) confirm reliable behavior across proxies.

Additionally, we compared the *p*O_2_ curve reconstructed by the URD model with reconstructions of the geological oxygenation history from other studies. For instance, when benchmarked against Mills’s reconstruction [30] using multiple geochemical indicators (δ^13^C, δ^34^S, etc.) for the Phanerozoic (Fig. S6), our results showed general consistency with previous study. Besides, by comparing with the reconstructions by Chen *et al.* [6] using igneous rock records (Fig. S7), we discussed the similarities and differences in *p*O_2_ changes during the three major periods of atmospheric oxygenations: the GOE, the NOE, and the Paleozoic Oxygenation Event. These comparisons demonstrate both consistency and complementarity across the findings, highlighting the importance and growing necessity of developing robust big-data methodologies to provide integrated insights from multiple disciplines in paleoenvironmental reconstruction (see Supplementary material for details).

Finally, although we adopted only limited number of *p*O_2_ labels (26 data points reconstructed from geochemical proxies and models), the reconstructed *p*O_2_ curve still risks a strong dependence on these labels. Therefore, we performed principal component analysis on the 25-dimensional Mn mineral feature space, with the top three principal components (PC1, PC2, and PC3) cumulatively explaining 71.4% of the variance (Fig. S8). The temporal variations of these principal components show strong coherence with the reconstructed *p*O_2_ curve. More importantly, all three principal component curves exhibit a marked rise, closely mirroring the timing and direction of the *p*O_2_ rise in our model reconstruction during the GOE (2.5 to 2.4 Ga). This demonstrates that the reconstruction of *p*O_2_ is not decisively influenced by the label, but modulated by the varied Mn mineral features in the dataset.

### Reconstructed five evolutionary stages of *p*O_2_ throughout 4.0 Gyr

**Figure 3.**
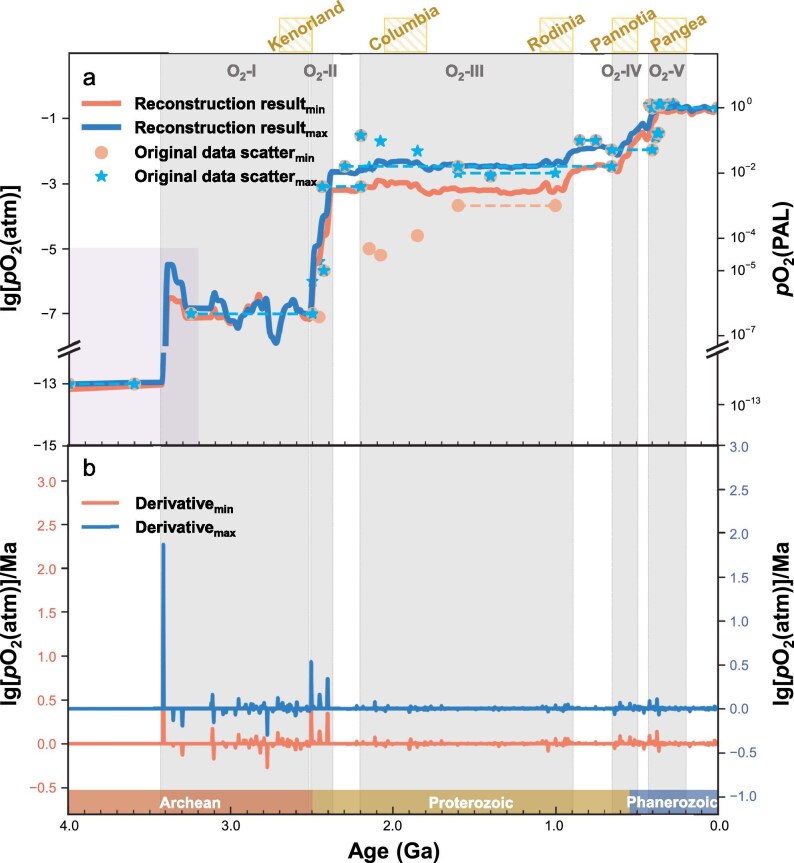
Reconstructed results of Earth’s atmospheric O_2_ levels (*p*O_2_) in the logarithm coordinate (a) and their first-order derivative with respect to time (b). The circular and star symbols show the minimum and maximum oxygen levels referred from literatures (Table S2), respectively. The two curves represent the minimum and maximum reconstructed results, respectively. Note that Fig. 3a uses nonequidistant right Y-axis. The curves in Fig. 3b correspond to the Y-axis of the same color. The five evolution stages of reconstructed *p*O_2_ are labeled with gray bands. The intervals in which Earth’s major supercontinents consolidated [49–52] are marked with yellow rectangles filled with slashes. The reconstruction accuracy in the purple region is limited by the sparseness of the Mn mineral data with ages before 3.2 Ga. The unit of *p*O_2_ is standard atmospheric pressure (atm), 1 PAL = 2.1 × 10^−1^ atm.

The logarithmic value of reconstructed *p*O_2_ (i.e. lg[*p*O_2_]) was shown in Fig. 3a, and we further calculated and summarized the changing rate of lg[*p*O_2_] at different stages (Table S7). Given the changing tempo and abrupt points of the rising rate, the specific time nodes of some major oxygenation events, such as the GOE, could be determined according to the first-order derivative of lg[*p*O_2_] over time (Fig. 3b). At least five significant periods in the evolution history of Earth’s *p*O_2_ are suggested by our analysis. Stage O_2_-I, 3.4 to 2.5 Ga, saw a rise in *p*O_2_ from 10^−13^ to >10^−7^ atm, followed by oscillations between ∼10^−8^ to 10^−7^ atm. During Stage O_2_-II, 2.5 to 2.4 Ga, *p*O_2_ rapidly increased from ∼10^−6^ to 10^−3^ atm. This second stage of Earth’s *p*O_2_ evolution can be equated to the GOE because the redox state of Earth’s atmosphere was irreversibly changed after that. According to the URD model, the *p*O_2_ was already above 10^−5^ PAL by 2.45 Ga, some 250 million years earlier than current estimate from the permanent loss of the mass-independent fractionation of sulphur isotopes (MIF-S) signal (2.2 Ga) [8].

Stage O_2_-III, 2.2 to 0.9 Ga, saw a small fluctuation in *p*O_2_ within the range of 6.6 × 10^−4^ to 4.1 × 10^−3^ atm before 0.95 Ga, and an immediate increase to 2.4 × 10^−3^ (minimum) to 0.01 atm (maximum) at around 0.9 Ga, encompassing Earth’s history from the assembly of Columbia to the Rodinia supercontinent. During Stage O_2_-IV, from 0.65 to 0.5 Ga, *p*O_2_ rapidly increased, approaching 2.1 × 10^−2^ atm—a level high enough to potentially enable the evolution of relatively high *p*O_2_-demand metazoans [31,32]. Finally, in Stage O_2_-V, from 0.43 to 0.2 Ga, *p*O_2_ rapidly increased to near present level (2.1 × 10^−1^ atm) during the consolidation of the Pangea supercontinent. The gentle increasing tendency of *p*O_2_ during 2.2 to 0.9 Ga is consistent with the relatively stable frequency of new mineral deposition during this period (Fig. 1 and Figs S9 and S10).

Notably, the rises in the occurrence frequency and periodic explosions of Mn mineral species (Fig. 1a) were roughly consistent with the predicted general trend of rising *p*O_2_ (Fig. 3), and the dynamic changes in both were tied to plate tectonics (Fig. 3 and Table S7). This observation is consistent with Hummer’s [21] reports that the maxima in Mn mineralization occurred in close proximity to supercontinent assembly events. Therefore, the evolution of *p*O_2_ on billion-year timescales was not simply a localized surface process, but was influenced by global-scale and deep processes such as plate tectonics that brought significant changes in the species and volumes of materials from the Earth’s interior [2,21]. As evidence, in the reconstructed global paleogeographic maps of Mn minerals’ distribution over the past 545 Ma (see Supplementary Video), we observed an intensified production of new Mn mineral species along convergent margins, such as during the convergence of Pannotia and Pangea supercontinents (Fig. S11), indicating the enhanced Mn mineralization by accumulation of appreciable Mn and reconfiguration of physicochemical conditions through plate tectonics.

### Oxygenation timing and tempo linked to plate tectonics and biological activities: evidence from Mn minerals

The URD deep-learning model demonstrates the potential for reconstructing historical *p*O_2_ using a global-scale, deep-time dataset of Mn minerals. Through the URD model, we further identified the set of Mn minerals most correlated with *p*O_2_ at the corresponding age (Table S8). By analysing the mineral genesis associated with each set, we discovered several factors that influenced the extent and rate of Earth’s oxygenation.

#### Stage O_2_-I and II (3.4 to 2.45 Ga): rapid oxygenation associated to plate tectonics

As shown in Figs 1 and 3, the first observable increase in *p*O_2_ appears to have occurred during Stage O_2_-I (3.4 to 2.5 Ga), with several noticeable fluctuations of *p*O_2_ between 10^−8^ and 10^−6^ PAL, accompanied by an increasing deposition frequency of Mn^III/IV^ oxides, C^IV^ carbonates, Nb^III^ niobates, and Ta^V^ tantalates. The time interval in which these relatively oxidized minerals appeared falls within the span now estimated by biologists and paleontologists for the emergence of oxygenic photosynthesis [33–37]. The number of redox-insensitive C- and P-containing minerals also showed a discernible increase during this period (Fig. S9). Notably, those Mn^III/IV^ oxides that came after 3.1 Ga shared both structural and functional similarities with the Mn_4_CaO_5_ cluster in oxygen-evolving complex (OEC) of photosystem II (PSII), and were highly efficient water oxidation catalysts [38–42], pointing to a possible abiotic pathway of water splitting to produce O_2_ [40,42].

Although *p*O_2_ exhibited frequent fluctuations from 3.4 to 2.5 Ga, they never fell below 10^−5^ PAL after about 2.45 Ga (Fig. 3a). We note that higher O_2_ production rates in the Archean and Proterozoic Eons occurred during the breakup of Kenorland and Rodinia supercontinents (Fig. 3 and Table S7). Besides Mn oxides, our analysis identified some hydrothermally or magmatically sourced Mn^II^ minerals as strongly correlated with *p*O_2_ trends (Fig. 1 and Table S8). This correlation indicates that atmospheric oxygenation in the Archean Eon was linked to deep Earth processes that might have significantly modulated the O_2_ source–sink balance [43]. Further, the increased Mn flux from the breakup of supercontinents could have facilitated the extensive assembly of PSII, given Mn’s essential role in the oxygen-evolving center. This process may have been a key driver of the first explosion of *p*O_2_ during the 2.5 to ∼2.4 Ga GOE (Fig. 3 and Table S7).

#### Stage O_2_-III (2.2 to 0.9 Ga): moderately active evolution of Mn minerals and *p*O_2_

We also observe a protracted steady increase of *p*O_2_ at a very low rate during Stage O_2_-III (Fig. 3 and Table S7). The Mn minerals with the highest correlation with *p*O_2_ during this stage (2.2 to 0.9 Ga) include Mn oxysalts and layered or tunneled Mn^III/IV^ (hydr)oxides, mostly from supergene geochemical processes (Table S8). The transformation from Columbia to Rodinia supercontinent may have involved relatively limited continental dispersion and a slight reconstruction [44]. Consequently, relatively stable continents brought steady Mn fluxes (as a functional element for life) and nutrients (e.g. P, as a nutrient element for life) through weathering, which could be conducive to the expansion of the ecological niche of oxygenic photosynthetic organisms and the increase of oxygen source. Moreover, due to the lack of large tectonic events, reducing materials from the deep Earth (i.e. the oxygen sink) was inadequate to suppress a rise in atmospheric oxygen. With the gentle increase of *p*O_2_, redox-sensitive Mn minerals underwent oxidative transformation and evolved some low-temperature supergene minerals such as Mn oxides and oxysalts (Fig. 1). These mineral records provided evidence for the moderately active evolution of Mn minerals and near-surface environments including *p*O_2_ even during the period of diminished orogeny.

#### Stage O_2_-IV and V (0.65 to 0.2 Ga): variations of *p*O_2_ linked to relative changes in the supply–demand levels of bioavailable nutrient and functional elements

Unlike the rapid increase in *p*O_2_ prior to 0.9 Ga, particularly during the breakup of supercontinents such as Kenorland and Rodinia, the sustainable increase of *p*O_2_ to the present level occurred during the assembly of Pannotia (0.65 to 0.5 Ga) and Pangea (0.43 to 0.2 Ga). This change is generally attributed to the balance between O_2_ sources and sinks [43]. In the early stage (4.0 to 0.9 Ga, before the NOE) with low *p*O_2_, there was very limited Mn flux from continental weathering, with both Mn and other nutrients essential for oxygenic photosynthetic organisms primarily sourced from deep Earth. Therefore, the breakup of supercontinents contributed a major Mn flux to early photosynthetic cyanobacteria. The increasing species diversity and solubility of Mn minerals after 0.9 Ga indicated higher Mn^2+^ concentrations in oceans or sedimentary environments, providing sufficient Mn sources for further flourishing oxygenic photosynthetic organisms (Fig. 1 and Fig. S1). As *p*O_2_ increased to high levels, there was also a substantial increase in the flux of Mn and essential nutrients derived from continental weathering, rendering them no longer defining factors for oxygenic photosynthesis. Notably, supercontinent assembly during this time period not only released essential nutrients and functional elements that continuously contributed to increasing O_2_ sources [43], but also significantly diminished those reducing materials from the deep Earth as potential O_2_ sinks. Plate tectonics can therefore play different roles in regulating Earth’s oxygenation events, depending on relative changes in the supply-demand levels of bioavailable Mn and essential nutrient elements.

The Mn mineral sets most closely linked to *p*O_2_ after 0.65 Ga mainly include low-temperature weathering products with various redox-sensitive elements (i.e. Cr, V, U), such as reynoldsite (Pb_2_Mn^IV^_2_O_5_(Cr^VI^O_4_)), santafeite ((Ca, Sr, Na)_3_(Mn^II^, Fe^III^)_2_Mn^IV^_2_(V^V^O_4_)_4_ (OH, O)_5_·2H_2_O), and redcanyonite ((NH_4_)_2_Mn^II^[(U^VI^O_2_)_4_O_4_(SO_4_)_2_](H_2_O)_4_) (Table S8). In this way, the accumulation of *p*O_2_ enabled Mn redox activity and rapid cycling in Earth’s near-surface system. In addition, some hydrothermal or metamorphic minerals contributed to *p*O_2_, as represented by epidote (khristovite, CaCe^III^(MgAlMn^II^)[Si_2_O_7_][SiO_4_]F(OH)), garnet (momoiite, Mn^II^_3_V^III^_2_(SiO_4_)_3_), and spinel (vuorelainenite, Mn^II^V^III^_2_O_4_) group minerals (Table S8), which provided an alternative supply of Mn and other essential nutrient elements via orogenesis or hydrothermal activity into the oceans or sedimentary environments. The increase in the total flux of functional and nutrient elements required by photosynthetic organisms to increase *p*O_2_, is a necessary prerequisite for the explosion of Mn-centered oxygenic photosynthesis.

We conclude that the evolutionary characteristics of global Mn mineral diversification driven by both plate tectonics and biological activities may preserve the evolutionary direction and tempo of Earth’s oxygenated atmosphere. The positive contribution of several hydrothermally-, magmatically- and metamorphically-sourced Mn silicates and sulfides in plate tectonics events (Table S8) to each abrupt increase in *p*O_2_, suggests that Mn, as a key functional element in biological antioxidant and oxygenic action, may have partially influenced the Earth’s oxygenation process. Tectonic events also bring oxygenic cyanobacteria from deep Earth or continents to the euphotic zone of oceans by influencing ocean circulation, opening up previously inaccessible ecological niches and eventually causing the expansion and eruption of cyanobacteria to contribute to rapid increases of *p*O_2_.

## CONCLUSIONS

In summary, this study reconstructs Earth’s atmospheric oxygenation history by decoding the coevolutionary relationships between *p*O_2_ and the phylogenetic histories of Mn minerals over nearly 4.0 Gyr. Our URD deep‑learning network integrates high‑dimensional, time‑varying mineralogical features via an RNB‑MLP block to produce a continuous, uniform-scale *p*O_2_ curve that spans the entire geological record. The reconstruction demonstrates high predictive accuracy (MAPE < 0.2, MSE < 0.2, and *R^2^* > 0.96) and reveals some new features of Earth’s oxygenation trajectory: frequent *p*O_2_ fluctuations from 3.4 to 2.5 Ga, an elevated *p*O_2_ exceeding 10^−5^ PAL as early as 2.45 Ga, a protracted and monotonic *p*O_2_ rise through the Paleoproterozoic–Mesoproterozoic, a distinct coupling between Neoproterozoic oxygenation pulses and supercontinent cyclicity, and finally reaching modern levels around ∼420 Ma. By identifying age-specific Mn mineral assemblages most strongly linked to *p*O_2_, we provide global-scale mineralogical evidence that the timing and tempo of Earth’s oxygenation were tightly coupled to plate tectonics and biosphere evolution.

These findings establish mineral informatics as a novel approach for paleoenvironmental quantification, demonstrating how deep-learning-driven analysis of mineralogical big data can reveal coevolutionary dynamics among Earth’s tectonic, mineralogical, and atmospheric systems. The presented methodology is readily transferable to other paleoenvironmental parameters and provides a generalizable framework for investigating the deep-time coevolution of the lithosphere, hydrosphere, and biosphere.

## METHODS

The complex physicochemical changes and interactions between deep-time Mn minerals and atmospheric O_2_, and the varying Mn mineral sets at different geologic ages, along with the time sensitivity of the coevolutionary process, make it difficult to reconstruct the relationship between Mn minerals and *p*O_2_. Even deep-learning models, with large parameter space and full supervision signals, would fall into the dilemma of a URD caused by the above problems. To address those problems and establish an efficient mapping from Mn minerals to *p*O_2_, at any age between 0 to 4.0 Ga, we proposed a deep-learning architecture, namely URD model.

### Construction of Mn mineral feature dataset

The Mn mineral data cover all 5768 dated Mn mineral samples across 476 Mn mineral species certified by International Mineralogical Association from both RRUFF (https://rruff.info/evolution) and Mindat (https://mindat.org/) database (up to December 2021), over the geological period of 0.0 to 4.0 Ga. However, since the Mineral Evolution Database reports most of mineral ages in the form of age ranges rather than age points, we conducted an interval strategy (details in subsection *URD model*) to minimize the negative impacts caused by the data uncertainty. Besides, in order to further ensure the geological credibility (e.g. the impact of secondary alteration effects) of the data, we conducted careful data cleansing with screening rules. Screening rules include the following: (i) Mineral samples that are directly dated by themselves or specific elements contained in themselves, or are primary minerals with ages assigned by primary host rocks, are considered as good data points. (ii) Nondirectly dated secondary mineral samples (including the products of aqueous low-temperature alteration or subaerial oxidative hydration/weathering, and subaerial alteration) older than 100 Ma are considered for screening, while those younger than 100 Ma are retained. (iii) For the data points to be screened in (ii), those with a minimum age older than the lower age limit of their secondary process, are directly removed from the dataset.

In particular, there are three reasons for retaining the mineral samples younger than 100 Ma as follows: (i) For minerals with a minimum age <100 Ma, the age uncertainty caused by secondary processes should be within 100 Ma. (ii) The retained mineral samples (that may be related to secondary processes) make up only a small portion of all minerals samples with age <100 Ma, thus making the impact of secondary processes quite limited. (iii) Our URD model adopts a ‘pretraining’ + ‘fine-tuning’ training approach. During pretraining, high-precision ice core data with ages concentrated around 100 Ma are utilized, resulting in higher prediction accuracy for samples closer to 100 Ma in the complete dataset. We further verified the accuracy of the model with test datasets including ice core data (MSE < 0.08, MAPE < 0.05, and *R*^2^ > 0.99), indicating the good performance of the model on dataset that combines these retained mineral samples.

According to multidimensional mineral crystal chemistry characteristics and occurrences, which had associated geologic age of all Mn mineral samples, we summarized 25-dimensional characters (Table S1) to represent each Mn mineral sample. The 25-dimensional characters include six types of mineralogical features: geologic age, chemical composition, key elements, Mn valence states, structural symmetry, and chemical classifications. Especially, ‘chemical classifications’ are represented by a group of numbers from 1 to 17 (i.e. one dimension) for dimensionality reduction in the algorithm. Besides, to increase the identification features between minerals, it is necessary to add some ‘key elements’ such as phosphorus (P), chromium (Cr), and vanadium (V) although there are phosphate, chromate, and vanadate in ‘chemical classification’. For example, some minerals belong to silicates but also contain P, including lipuite and yoshimuraite. Many Mn minerals inherently incorporate some key redox-sensitive elements (Fe, Cr, Ce, V, U, Mo, etc.), within their crystal structures. Consequently, the features of these redox-sensitive proxies are also integrated into the Mn mineral dataset. Finally, a deep-time Mn mineral dataset containing a total of 5768 × 25 = 144 200 features was constructed. In order to create the scatter diagram of Mn mineral evolution over 4.0 Ga, all 5768 dated Mn mineral samples were marked according to their maximum geologic age and Mn valence states, and the classification of each sample was plotted on the vertical axis (Fig. 1).

We also collected the longitude and latitude information of Mn minerals from RRUFF (https://rruff.info/evolution) and mindat.org, and constructed a video of the global distribution of Mn minerals over the past 545 Ma (see Supplementary Video) through Gplates 2.3.0 and data of paleographic reconstruction from Scotese [45].

### Construction of atmospheric O_2_ level dataset

We collected 552 *p*O_2_ data across deep time, including 526 groups of ice core records data (0.0 to 0.78 Ma) and 26 groups of *p*O_2_ data based on geochemical proxies and models (0.0 to 4.0 Ga). It is worth noting that, in order to avoid the one-sidedness of one single proxy, we try to extract *p*O_2_ data as many as possible from different proxies or models (Table S2). Each group of *p*O_2_ data includes three characteristics: age, the maximum *p*O_2_, and the minimum *p*O_2_. We use O*_T_*_1_ and O*_T_*_2_ to represent the augmented data from ice core records and geochemical proxies and models, respectively. Considering the high data density of dataset O*_T_*_1_, we first pretrained the model on dataset O*_T_*_1_ and then performed migration learning on both dataset O*_T_*_1_ and O*_T_*_2_. The two-stage training strategy enables our model to quickly capture the latent mapping between Mn minerals and *p*O_2_, and extend the mapping to the whole geological period from 0.0 to 4.0 Ga.

### URD model

#### Problem formulation

Let *E* represent the complex coevolution relationship between Mn minerals and *p*O_2_. Mapping from Mn minerals to *p*O_2_ could be modeled as


(1) \begin{eqnarray*}
{C}_t = E(M),
\end{eqnarray*}

where *C_t_* represents *p*O_2_ at age *t, M* = {*M*_1_, *M*_2_,…, *M_m_*} represents the set of Mn minerals with *m* as the number of Mn mineral samples at age *t*. In order to build an effective model *E* via deep learning, three pivotal problems need to be addressed.

The first problem is recoupling relationships among contemporaneous Mn minerals. *p*O_2_ at any age, which was affected by a set of strongly coupled Mn minerals, represents the result of complex chemical changes and interactions of various Mn minerals over long-time intervals. To address this problem, we decomposed model *E* into two steps: (i) Function *E_k_* models the influence factor of Mn mineral sample *M_k_* on *p*O_2_; (ii) Function *D* fuses the influence factor under age *t* to obtain the *p*O_2_ value:


(2) \begin{eqnarray*}
&& {C}_t = D\left[ p(1,t){E}_1({M}_1),p(2,t){E}_2({M}_2),..., \right. \\
&&\qquad \left. p(m,t){E}_m({M}_m) \right],
\end{eqnarray*}

where *p*(*k, t*) represents whether the geologic age of Mn mineral *M_k_* is at age *t*. Since a Mn sample is represented by a period $[ {T_k^{\min },T_k^{\max }} ]$ when $T_k^{\min } \le t \le T_k^{\max }$ is satisfied, the value of *p*(*k, t*) is 1, otherwise *p*(*k, t*) equals 0. Through Equation (2), the evolution regulation of different Mn mineral samples could be traced by specific and interpretable *E_k_*(*M_k_*).

The second problem comes from the varying Mn mineral species corresponding to different ages, leading to unequal feature matrix sizes of Mn mineral samples at different ages. *p*O_2_ at each age *t* is affected by a set of Mn minerals with very different mineral numbers ranging from 3 to 683. Thus, sizes of feature matrices used as input of networks would apparently change. To address this problem, a unified model *E*_CM_ with adaptive shared parameters was constructed to compute the influence factor of each Mn mineral on *p*O_2_:


(3) \begin{eqnarray*}
&& {C}_t = D\left[ p(1,t){E}_{{\mathrm{CM}}}({M}_1),p(2,t){E}_{{\mathrm{CM}}}({M}_2),..., \right. \\
&&\qquad \left. p(m,t){E}_{{\mathrm{CM}}}({M}_m) \right].
\end{eqnarray*}

In this way, the deep-learning network could be trained by iteratively updating the shared parameters in *E*_CM_. Furthermore, mineral records in geological databases inevitably suffer from the issue of preservation bias. The mechanism of adaptive shared parameters, however, allows mineral samples of arbitrary size to be used as inputs with consistent length, thereby reducing the impact of sample size differences in different periods (i.e. varying amounts of minerals preserved at different periods) on model predictions.

The third problem, disaccord labels, stems from the wide range of geologic ages of Mn mineral occurrences, resulting in inexplicit correspondence between Mn minerals and *p*O_2_. The coevolution process of Mn minerals and atmospheric O_2_ is sensitive to time, while the Mn mineral feature dataset lacked features to reflect this process. As a result, a number of ages shared repeat mineral characteristics but indicated different *p*O_2_ values. To resolve this problem, we let ${M}_k = {M}_k[u] \oplus {M}_k[t],k \in [1,n]$. *M_k_*[*u*] represent time-invariant properties of the mineral *M_k_*, which are directly extracted from the Mn feature dataset. *M_k_*[*t*] represents time-variant evolution properties of the mineral *M_k_*. By introducing *M_k_*-containing features of evolution, the explicit correspondence between Mn minerals and *p*O_2_ at age *t* could be expressed as Equation (4), which can be modeled by deep-learning methods:


(4) \begin{eqnarray*}
{C}_t = D_{k = 1}^m\left[ {p(k,t){E}_{CM}({M}_k[u],{M}_k[t])} \right].
\end{eqnarray*}

A deep-learning model called URD to model Equation (4) was designed to solve the above three problems and thus to establish the continuous coevolution relationship between Mn mineral and *p*O_2_. The URD model (Fig. 2b) was composed of three blocks: feature extraction block, parallel inference block RNB-MLP, and logarithmic loss function. Furthermore, in comparison to traditional methods, URD addressed concerns regarding the potential influence of Mn minerals with little correlation to *p*O_2_ by leveraging the deep-learning model’s intrinsic ability to automatically select useful features. The URD model effectively disregarded those less pertinent to the target while prioritizing more critical features. Specifically, the URD model dealt with Mn mineral features in parallel and adjusted the model parameters using gradient backpropagation after all Mn mineral features had been processed. This design made the Mn minerals that contributed less to *p*O_2_ have a smaller gradient, and then had a more subtle impact on the model update. With this strategy, we ensured that the model could effectively utilize useful Mn mineral samples while ignoring those that had little correlation with *p*O_2_.

#### Feature extraction block

The feature extraction block was designed to normalize the Mn mineral features at a specific age *t*, which would produce unique Mn mineral feature data with *p*O_2_ label. Age *t* and corresponding *p*O_2_, labels were randomly extracted from O*_T_*_1_ or O*_T_*_2_. The feature extraction block collected all *m_t_* minerals *M* = {*M*_1_, *M*_2_,…, *M_m_*} dated at age *t* from the Mn mineral dataset, represented by a feature matrix {*M_k_*[*u*]} with dimensions of *m_t_* × 25. Taking into account the evolution in Mn mineral samples over time, the feature extraction block created a token *M_k_*[*t*] associated geologic age for each Mn mineral sample in a normalization layer. The normalization layer combined age *t* with geological period $[ {T_k^{\min },T_k^{\max }} ]$ of Mn mineral sample *M_k_*, which produced the corresponding tokens at age *t* as


(5) \begin{eqnarray*}
\textit{Token}_{k1}(t) = \frac{{t - T_k^{\min }}}{{t + \varepsilon }},
\end{eqnarray*}

(6) \begin{eqnarray*}
\textit{Token}_{k2}(t) = \frac{{T_k^{\max } - t}}{{T_k^{\max } + \varepsilon }},
\end{eqnarray*}

where $\varepsilon = 0.01$ was a constant to avoid zero denominator. By matching {*M_k_*[*u*]} and a two-dimensional feature set as {*M_k_*[*u*]|*M_k_*[*t*]=[*Token_k_*_1_(*t*), *Token_k_*_2_(*t*)]}, the feature extraction block created the *n_t_* × 27 dimensional features to represent input data of RNB-MLP. In this way, we constructed a unique Mn mineral feature data with *p*O_2_ label following chronological order, and resolved the disaccord label problem of *p*O_2_. For all these features, we performed SHapley Additive exPlanations analysis and identified 10 characteristic parameters with the highest influence on the maximum atmospheric oxygen concentration near the NOE (Fig. S12).

#### Parallel inference block RNB-MLP

A four-layer RNB-MLP inference block was built to process input data with the unequal size of feature matrix of Mn minerals, and decouple the recoupled Mn samples. RNB-MLP treated features from each mineral sample as a group of parallel inputs, which were abstracted as parallel transposition layers. To summarize, it took *n_t_* as a batch dimension to deal with the significant change in number of Mn mineral samples at different age *t*. As a result, rows of feature matrix corresponding to different Mn mineral samples shared the same network parameters. The transposition layer effectively reduced parameters, which thus suppressed the possible network overfitting. Inference layers of RNB-MLP were composed of a full-connect block [46] with Leaky Rectified Linear Unit (Leaky-ReLU) as the activation function. Residual structure [47] was added to simplify optimization of the network. In addition, the batch normalization layer [48] was discarded to avoid interactions between Mn mineral samples. The network output had *n_t_* × 2 dimensions, representing the correlation coefficient of each input Mn mineral sample with *p*O_2_ over age *t*, respectively.

#### Logarithmic loss function

The loss function was leveraged as an association restriction between Mn minerals and *p*O_2_ to optimize the network parameters. In the loss function, the prediction error of network at age *t* was used as constraint. We incorporated logarithmic operations in URD’s loss function and made allowance for exponentially scaled changes in *p*O*_2_. predict_j_* and *label_j_* were used to represent the final predicted output of URD and the label of *p*O*_2_*. The loss function was computed as follows:


(7) \begin{eqnarray*}
{{\textit predic}}{{{t}}}_j = \frac{1}{{{m}_j}}\sum\limits_{i = 0}^{{m}_j} {{{ou}}{{{t}}}_{{j}_i}} ,
\end{eqnarray*}

(8) \begin{eqnarray*}
{\mathrm{Loss}} = \frac{1}{h}\sum\limits_{j = 0}^h {{{\left( {{{\textit predic}}{{{t}}}_j - V\left( {{{\log }}_{10}\!\left( {{{labe}}{{{l}}}_j} \right)} \right)} \right)}}^2,}
\end{eqnarray*}

where *h* was the size of a batch and ${{{ou}}{{{t}}}_{{j}_i}}$ was the contribution of Mn mineral sample *i* at age *j. V* was an axis spacing handler, defined as follows:


(9) \begin{eqnarray*}
V(x) = - \ln ( - x).
\end{eqnarray*}

The relation between ${{{ou}}{{{t}}}_{{j}_i}}$ and predict result was computed as:


(10) \begin{eqnarray*}
\displaystyle\frac{{\partial {{10}}^{{V}^{ - 1}(\textit{predict}_j)}}}{{\partial ou{t}_{{j}_i}}} &= \ln 10 \cdot {10}^{ - {{\mathrm{e}}}^{ - \textit{predict}_j}} \\
& \cdot\, \displaystyle\frac{{{{\mathrm{e}}}^{ - \textit{predict}_j}}}{{{m}_j}},
\end{eqnarray*}

where $\partial $ is the partial differential notation and *V*^−1^ is inverse function of *V*. Since the derivative is always positive, the final predicted *p*O_2_ is positively correlated with ${{{ou}}{{{t}}}_{{j}_i}}$. For *p*O_2_ benchmarks at different age *j*, the criterion of whether effects from the Mn mineral sample *i* on *p*O_2_ is promoting or inhibiting, was optimized as follows: if ${{{ou}}{{{t}}}_{{j}_i}} > \frac{1}{{{m}_j}}\sum\nolimits_{i = 0}^{{m}_j} {{\textit out}_{{j}_i}} $, Mn mineral sample *i* has a promoting effect on *p*O_2_ at age *j*, and vice versa.

#### Implementation

Our URD model was developed and implemented using PyTorch 1.10.0 on a platform with one NVIDIA GeForce GTX 1650 SUPER graphics card. The training strategy included two stages: ‘pretraining’ and ‘migration training’. We randomly selected 75% groups from *p*O_2_ dataset as training sample while treating the remaining 25% groups as verification sample. In the pretraining stage, URD was trained on the data from O*_T_*_1_ by an Adam optimizer, and the learning rate was 0.01. In the migration training stage, URD was trained on the data from O*_T_*_1_ and O*_T_*_2_. The initial learning rate of migration training stage was 0.01, which automatically decreased following the increase of the training epoch. The loss curve and residual curve are shown in Fig. S13. The pretraining stage enables the model to iterate from a position with a small loss and quickly converge within 100 iterations during migration training.

#### Constructed atmospheric O_2_ data presentation

The logarithmic coordinate (Fig. 3a) was used to show the relative fluctuation trend of *p*O_2_ (details showed in Fig. S14). Moreover, the first-order derivative of lg[*p*O_2_] with respect to *t* was plotted (Fig. 3b) to show the sudden change of *p*O_2_, based on Equation (11):


(11) \begin{eqnarray*}
f^{\prime}({t}_0) = \frac{{{\rm d}f}}{{{\rm d}t}} = \mathop {\lim }\limits_{\Delta t \to 0} \frac{{f({t}_0 + \Delta t) - f(t)}}{{\Delta t}}.
\end{eqnarray*}

## Supplementary Material

nwag230_Supplemental_Files

## Data Availability

Mn mineral characteristic data and atmospheric oxygen concentration reconstruction results are publicly available at https://github.com/a-Fomalhaut-a/Mn_O. The code used in this paper including the trained URD model is available online at https://github.com/a-Fomalhaut-a/Mn_O. We also provide a demo by which users could reconstruct atmospheric oxygen concentrations at any age by inputting a set of contemporaneous manganese minerals.

## References

[1] Catling D, Zahnle K. The Archean atmosphere. Sci Adv 2020; 6: eaax1420. 10.1126/sciadv.aax1420 32133393 PMC7043912

[2] Lyons TW, Diamond CW, Planavsky NJ et al. Oxygenation, life, and the planetary system during Earth’s middle history: an overview. Astrobiology 2021; 21: 906–23. 10.1089/ast.2020.2418 34314605 PMC8403206

[3] Rye R, Holland HD. Paleosols and the evolution of atmospheric oxygen: a critical review. Am J Sci 1998; 298: 621–72. 10.2475/ajs.298.8.621 11542256

[4] Claire MW, Kasting JF, Domagal-Goldman SD et al. Modeling the signature of sulfur mass-independent fractionation produced in the Archean atmosphere. Geochim Cosmochim Acta 2014; 141: 365–80. 10.1016/j.gca.2014.06.032

[5] Johnson JE, Gerpheide A, Lamb MP et al. O_2_ constraints from Paleoproterozoic detrital pyrite and uraninite. GSA Bull 2014; 126: 813–30. 10.1130/B30949.1

[6] Chen G, Cheng Q, Lyons TW et al. Reconstructing Earth’s atmospheric oxygenation history using machine learning. Nat Commun 2022; 13: 5862. 10.1038/s41467-022-33388-5 36195593 PMC9532422

[7] Farquhar J, Bao H, Thiemens M. Atmospheric influence of Earth’s earliest sulfur cycle. Science 2000; 289: 756–8. 10.1126/science.289.5480.756 10926533

[8] Poulton SW, Bekker A, Cumming VM et al. A 200-million-year delay in permanent atmospheric oxygenation. Nature 2021; 592: 232–6. 10.1038/s41586-021-03393-7 33782617

[9] Anbar AD, Duan Y, Lyons TW et al. A whiff of oxygen before the great oxidation event? Science 2007; 317: 1903–6. 10.1126/science.1140325 17901330

[10] Frei R, Gaucher C, Poulton SW et al. Fluctuations in Precambrian atmospheric oxygenation recorded by chromium isotopes. Nature 2009; 461: 250–3. 10.1038/nature08266 19741707

[11] Crowe SA, Døssing LN, Beukes NJ et al. Atmospheric oxygenation three billion years ago. Nature 2013; 501: 535–8. 10.1038/nature12426 24067713

[12] Reinhard CT, Planavsky NJ, Robbins LJ et al. Proterozoic ocean redox and biogeochemical stasis. Proc Natl Acad Sci USA 2013; 110: 5357–62. 10.1073/pnas.1208622110 23515332 PMC3619314

[13] Stolper D, Bender M, Dreyfus G et al. A Pleistocene ice core record of atmospheric O_2_ concentrations. Science 2016; 353: 1427–30. 10.1126/science.aaf5445 27708037

[14] Yan Y, Brook EJ, Kurbatov AV et al. Ice core evidence for atmospheric oxygen decline since the Mid-Pleistocene transition. Sci Adv 2021; 7: eabj9341. 10.1126/sciadv.abj9341 34910502 PMC8673763

[15] Post JE . Manganese oxide minerals: crystal structures and economic and environmental significance. Proc Natl Acad Sci USA 1999; 96: 3447–54. 10.1073/pnas.96.7.3447 10097056 PMC34287

[16] Brusnitsyn A . Associations of Mn-bearing minerals as indicators of oxygen fugacity during the metamorphism of metalliferous deposits. Geochem Int 2007; 45: 345–63. 10.1134/S0016702907040039

[17] Bertine KK, Turekian KK. Molybdenum in marine deposits. Geochim Cosmochim Acta 1973; 37: 1415–34. 10.1016/0016-7037(73)90080-X

[18] Eary LE, Rai D. Kinetics of chromium (III) oxidation to chromium (VI) by reaction with manganese dioxide. Environ Sci Technol 1987; 21: 1187–93. 10.1021/es00165a005

[19] Ostrander CM, Johnson AC, Anbar AD. Earth’s first redox revolution. Annu Rev Earth Planet Sci 2021; 49: 337–66. 10.1146/annurev-earth-072020-055249

[20] Barling J, Arnold GL, Anbar A. Natural mass-dependent variations in the isotopic composition of molybdenum. Earth Planet Sci Lett 2001; 193: 447–57. 10.1016/S0012-821X(01)00514-3

[21] Hummer DR, Golden JJ, Hystad G et al. Evidence for the oxidation of Earth’s crust from the evolution of manganese minerals. Nat Commun 2022; 13: 960. 10.1038/s41467-022-28589-x 35181670 PMC8857192

[22] Thonis M, Burns RG. Manganese ore deposits and plate tectonics. Nature 1975; 253: 614–6. 10.1038/253614a0

[23] Yun S, Hwang H, Hwang G et al. Super-hydration and reduction of manganese oxide minerals at shallow terrestrial depths. Nat Commun 2022; 13: 1942. 10.1038/s41467-022-29328-y 35410458 PMC9001738

[24] Roy S . Sedimentary manganese metallogenesis in response to the evolution of the Earth system. Earth-Sci Rev 2006; 77: 273–305. 10.1016/j.earscirev.2006.03.004

[25] Spinks SC, Sperling EA, Thorne RL et al. Mesoproterozoic surface oxygenation accompanied major sedimentary manganese deposition at 1.4 and 1.1 Ga. Geobiology 2023; 21: 28–43. 10.1111/gbi.12524 36168296 PMC10087800

[26] Oliver N, Avramov AP, Nürnberg DJ et al. From manganese oxidation to water oxidation: assembly and evolution of the water-splitting complex in photosystem II. Photosynth Res 2022; 152: 107–33. 10.1007/s11120-022-00912-z 35397059

[27] Wang Z, Cai S, Chen G et al. Describe, explain, plan and select: interactive planning with LLMs enables open-world multi-task agents. Adv Neural Inf Process Syst 2023; 36: 34153–89.

[28] Swanson K, Wu E, Zhang A et al. From patterns to patients: advances in clinical machine learning for cancer diagnosis, prognosis, and treatment. Cell 2023; 186: 1772–91. 10.1016/j.cell.2023.01.035 36905928

[29] Mehrish A, Majumder N, Bharadwaj R et al. A review of deep learning techniques for speech processing. Inf Fusion 2023; 99: 101869. 10.1016/j.inffus.2023.101869

[30] Mills BJ, Krause AJ, Jarvis I et al. Evolution of atmospheric O_2_ through the Phanerozoic, revisited. Annu Rev Earth Planet Sci 2023; 51: 253–76. 10.1146/annurev-earth-032320-095425

[31] Sperling EA, Knoll AH, Girguis PR. The ecological physiology of Earth’s second oxygen revolution. Annu Rev Ecol Evol Syst 2015; 46: 215–35. 10.1146/annurev-ecolsys-110512-135808

[32] Catling DC, Glein CR, Zahnle KJ et al. Why O_2_ is required by complex life on habitable planets and the concept of planetary “oxygenation time”. Astrobiology 2005; 5: 415–38. 10.1089/ast.2005.5.415 15941384

[33] Jabłońska J, Tawfik DS. The evolution of oxygen-utilizing enzymes suggests early biosphere oxygenation. Nat Ecol Evol 2021; 5: 442–8. 33633374 10.1038/s41559-020-01386-9

[34] Boden JS, Konhauser KO, Robbins LJ et al. Timing the evolution of antioxidant enzymes in cyanobacteria. Nat Commun 2021; 12: 4742. 10.1038/s41467-021-24396-y 34362891 PMC8346466

[35] Satkoski AM, Beukes NJ, Li W et al. A redox-stratified ocean 3.2 billion years ago. Earth Planet Sci Lett 2015; 430: 43–53. 10.1016/j.epsl.2015.08.007

[36] Rosing MT, Frei R. U-rich Archaean sea-floor sediments from Greenland—indications of >3700 Ma oxygenic photosynthesis. Earth Planet Sci Lett 2004; 217: 237–44.

[37] Kirschvink JL, Kopp RE. Palaeoproterozoic ice houses and the evolution of oxygen-mediating enzymes: the case for a late origin of photosystem II. Philos Trans R Soc B-Biol Sci 2008; 363: 2755–65. 10.1098/rstb.2008.0024 PMC2606766 18487128

[38] Sauer K, Yachandra VK. A possible evolutionary origin for the Mn_4_ cluster of the photosynthetic water oxidation complex from natural MnO_2_ precipitates in the early ocean. Proc Natl Acad Sci USA 2002; 99: 8631–6. 10.1073/pnas.132266199 12077302 PMC124339

[39] Russell MJ, Allen JF, Milner-White EJ. Inorganic complexes enabled the onset of life and oxygenic photosynthesis. In: Allen JF (ed.). Photosynthesis. Energy from the Sun. Dordrecht: Springer, 2008, 1187–92. 10.1007/978-1-4020-6709-9

[40] Hocking RK, Brimblecombe R, Chang L-Y et al. Water-oxidation catalysis by manganese in a geochemical-like cycle. Nat Chem 2011; 3: 461–6. 10.1038/nchem.1049 21602861

[41] Suga M, Akita F, Hirata K et al. Native structure of photosystem II at 1.95 Å resolution viewed by femtosecond X-ray pulses. Nature 2015; 517: 99–103. 10.1038/nature13991 25470056

[42] Yang J, An H, Zhou X et al. Water oxidation mechanism on alkaline-earth-cation containing birnessite-like manganese oxides. J Phys Chem C 2015; 119: 18487–94. 10.1021/acs.jpcc.5b05989

[43] Campbell IH, Allen CM. Formation of supercontinents linked to increases in atmospheric oxygen. Nat Geosci 2008; 1: 554–8. 10.1038/ngeo259

[44] Tang M, Chu X, Hao J et al. Orogenic quiescence in Earth’s middle age. Science 2021; 371: 728–31. 10.1126/science.abf1876 33574211

[45] Scotese CR . PALEOMAP PaleoAtlas for GPlates and the PaleoData Plotter Program. PALEOMAP Project. https://www.earthbyte.org/paleomap-paleoatlas-for-gplates/ (7 August 2025, date last accessed).

[46] Tolstikhin IO, Houlsby N, Kolesnikov A et al. MLP-Mixer: an all-MLP architecture for vision. Adv Neural Inf Process Syst 2021; 34: 24261–72.

[47] Wu Z, Shen C, Van Den Hengel A. Wider or deeper: revisiting the ResNet model for visual recognition. Pattern Recognit 2019; 90: 119–33. 10.1016/j.patcog.2019.01.006

[48] Santurkar S, Tsipras D, Ilyas A et al. How does batch normalization help optimization? Adv Neural Inf Process Syst 2018; 31: 2483–93.

[49] Aspler LB, Chiarenzelli JR. Two Neoarchean supercontinents? Evidence from the Paleoproterozoic. Sediment Geol 1998; 120: 75–104. 10.1016/S0037-0738(98)00028-1

[50] Scotese CR . Late Proterozoic plate tectonics and palaeogeography: a tale of two supercontinents, Rodinia and Pannotia. In: Craig J (ed.). Global Neoproterozoic Petroleum Systems: The Emerging Potential in North Africa. London: Geological Society of London, 2009, 67–83.

[51] Cawood PA, Strachan RA, Pisarevsky SA et al. Linking collisional and accretionary orogens during Rodinia assembly and breakup: implications for models of supercontinent cycles. Earth Planet Sci Lett 2016; 449: 118–26. 10.1016/j.epsl.2016.05.049

[52] Mitchell RN, Zhang N, Salminen J et al. The supercontinent cycle. Nat Rev Earth Environ 2021; 2: 358–74. 10.1038/s43017-021-00160-0

