# Supplementary material for: From manganese mineral evolution history to atmospheric oxygen reconstruction
Source: Natl Sci Rev. 2026 Apr 17;13(11):nwag230. doi: 10.1093/nsr/nwag230 (PMC13251890; doi:10.1093/nsr/nwag230)
Supplement: nwag230_Supplemental_Files [file nwag230_supplemental_files.zip › Li et al. Revised Supplementary Materials.pdf]

Supplementary Materials of

From manganese mineral evolution history to atmospheric oxygen reconstruction

Yan Li[12]†*, Ziyi Zhuang[2]†, Xinran Xu[3]†, Rongzhang Yin[12]†, Yanzhang Li[12], Yadong Wang[3], Chunjiang Li[12], Yong Lai[1], Yanan Zhang[3], Huan Ye[12], Zhaoyang Hu[12], Anhuai Lu[2]*, Robert M. Hazen[4]*, Xiangzhi Bai[356]*

[1] SKLab-DeepMinE, MOEKLab-OBCE, School of Earth and Space Sciences, Peking University, Beijing 100871, China.

[2] Beijing Key Laboratory of Mineral Environmental Function, School of Earth and Space Sciences, Peking University, Beijing 100871, P.R. China.

[3] Image Processing Center, Beihang University, Beijing 102206, China.

[4] Earth and Planets Laboratory, Carnegie Institution for Science, Washington, DC 20015, USA.

[5] State Key Laboratory of Virtual Reality Technology and Systems, Beihang University, Beijing 100191, China.

[6] Advanced Innovation Center for Biomedical Engineering, Beihang University, Beijing 100083, China.

†These authors contributed equally to this work.
*Corresponding authors. Email: liyan-pku@pku.edu.cn, ahlu@pku.edu.cn, rhazen@ciw.edu, jackybxz@buaa.edu.cn.

Reconstruction performance evaluation

The proposed URD model is endowed with a feature extraction block and a parallel inference block specifically tailored for data with disaccord labels and unequal feature lengths, possessing broad application prospects in the geological field, especially for processing sparse data and reducing the risk of overfitting during training. We compared the URD model with several methods adapted to data with varying feature lengths. The Pad Zero method [1] selects 700 as the maximum number of input Mn mineral samples at a given age, and pads the empty dimensions with zeros. The Flag Identification method [2] takes the entire Mn mineral feature dataset as input and adds an additional dimension at the end of the feature matrix to label the state of the mineral. A flag value of 1 indicates the presence of the Mn mineral sample at a specific age, while a flag value of 0 indicates its absence. The Mean Feature method [3] computes the average features of all Mn mineral samples at the current age as input.

The comparative experimental results (Table S5) highlighted the exceptional performance of our URD model in managing such substantial variations in feature lengths. Unlike the Pad Zero and Flag Identification methods, which require a larger number of network parameters but yield poorer network performance, our URD model effectively adapted to new mineral sample data without requiring retraining. As the annual number of Mn mineral samples increases, overfitting due to excessive network parameters becomes more pronounced in the Pad Zero and Flag Identification methods. Despite the Mean Feature method having fewer parameters, it performed worse than our URD model. This disparity arises because the Mean Feature method struggles to directly capture the relationship between individual Mn mineral samples and pO_2. In comparison, the parallel inference block in the URD model facilitated the decoupling between Mn mineral samples. Moreover, the “time token” introduced in our URD model utilized the time-varying characteristic in the Mn feature extraction block. All of these improve the interpretability of the results, enabling focused analysis of specific Mn mineral samples or time points, thereby achieving a good performance in pO_2 reconstruction.

In addition, we conducted external validation using Mo mineral datasets as an independent redox proxy. Due to the limited availability of Mo mineral records for early Earth periods (Fig. S4), our comparative analysis focused on the 0−1.0 Ga timeframe. Remarkably, the URD model verified on Mo minerals produced oxygenation trends that align with those derived from Mn minerals, with only small variations in short-term fluctuations (Fig. S5). The quantitative performance metrics presented in Table S6 demonstrate comparable predictive accuracy between both proxy systems. This independent verification using Mo mineral datasets proved the robustness of the URD model. Importantly, the convergence of results from these distinct proxies strengthens the reliability of our pO_2 reconstruction.

Reconstruction comparison with previous studies

In order to evaluate our reconstruction results, as shown in Fig. S6, we compared the

Phanerozoic pO_2 curve established by Mills [4] using multiple geochemical indicators ($\delta^{13}C$, $\delta^{34}S$, etc.) with our reconstructed results. Overall, the two curves exhibited similar trends. In particular, during significant biological events such as the Great Ordovician Biodiversification Event (GOBE) and Early mammal evolution, associated with increased oxygen availability [5,6], our results accurately reflected these events with distinct oxygen peaks (highlighted by red arrows in Fig. S6b). In contrast, Mills' findings showed an opposing trend (Fig. S6a). Additionally, Fig. S6b revealed a short-term oxidation peak around 252 Ma, consistent with Newby's report [7] that identified a short-term ocean oxidation event during the end-Permian mass extinction (EPME) using $\delta^{205}Tl$ and other indicators. In the cases of the Cambrian Explosion and the beginning Age of Fishes, our results also showed similar increasing trends in pO_2 to those of Mills', although not in the form of distinct oxygen peaks. Recently, Stockey [8] found the similar major increase in pO_2 (accompanied by the major increase in marine dissolved $[O_2]$) during the mid-Palaeozoic through statistical learning analysis, which may suggest that O_2 played an important role in the Devonian radiation of fishes. Besides, Mills' curve showed finer fluctuations (especially during 550-400 Ma) due to integration of multiple indicators. Although they were interpreted as oxygen pulses, frequent and substantial changes of pO_2 seemed implausible. In contrast, our URD deep-learning model processed the Mn mineral dataset globally and non-linearly, resulting in a smoother curve that avoided potentially misleading jagged details. In summary, our reconstructed Phanerozoic pO_2 offers robust insights and demonstrates the reliability of the URD deep-learning model based on the Mn mineral dataset.

Besides, we also compared our reconstructed pO_2 curve with that of Chen et al. (2022) [9] using igneous rock records. Methodologically, both studies represent innovative, data-driven advances within respective disciplines (petrology and mineralogy) for paleoenvironmental reconstruction. So, their findings offer points of both consistency and complementarity with our findings. As illustrated in Fig. S7, we have compared our reconstruction with the three major periods of atmospheric oxygenation: the Great Oxidation Event (GOE), the Neoproterozoic Oxygenation Event (NOE), and the Paleozoic Oxygenation Event (POE). Notably, near the GOE and POE periods, our reconstructed curve exhibits a rapid increase in atmospheric O_2 level, whereas the curve in Chen et al. (2022) [9] is more subdued. Interestingly, recent studies on early marine oxygenation indicated the existence of O_2-rich environments around ~2.65−2.45 Ga, accompanied by the burial of Mn oxides [10,11]. This supports the possibility that the onset of the GOE may have preceded the broad 2.45−2.2 Ga constraint based on MIF-S isotopes, a timing more aligned with our reconstruction. However, the results in Chen et al. (2022) [9] during the NOE period showed a rapid increase in atmospheric O_2 level, which is less pronounced in our study. This discrepancy may be attributed to a relative scarcity of Mn mineral records around the NOE interval in our dataset (Fig. 1).

Principal component analysis (PCA) of Mn mineral features

To directly test whether the model's output is dictated by the labels or driven by the mineral data, we performed the Principal Component Analysis (PCA) on the 25-

dimensional Mn mineral feature space. The top three principal components (PCs), which cumulatively explain 71.4% of the total variance (PC1=46.8%, PC2=15.9%, PC3=8.7%), serve as an unbiased, data-derived summary of the major trends in Mn mineral evolution. As shown in Fig. S8, the temporal variations of PC1, PC2, and PC3 show strong coherence with the reconstructed pO_2 curve. During the GOE (2.5−2.4 Ga): All three principal components curves exhibit a marked rise, closely mirroring the timing and direction of the pO_2 rise in our model reconstruction. This indicates that the signal is present in the mineral data itself. In the Mid-Proterozoic (2.2−1.8 Ga): This period is characterized by particularly poor consistency among existing pO_2 labels, with deviations reaching 10^4 atm. Yet the O_2 curve predicted by our model is consistent with the variation of the principal component curve. This demonstrates that the reconstruction of pO_2 is not decisively influenced by the label, but modulated by the varied Mn mineral features in the dataset.

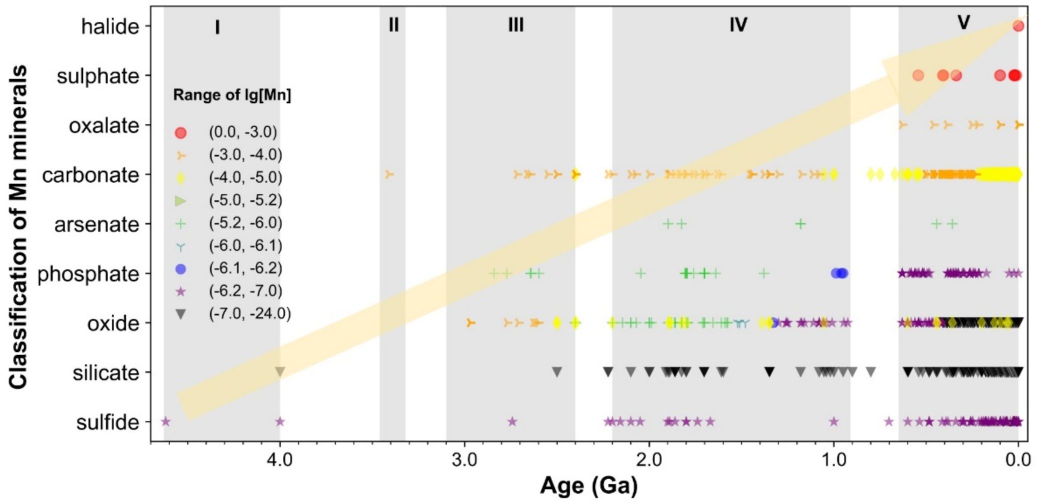

Figure S1. Solubility of some major Mn minerals versus their earliest age. As there are few studies on direct measurement of mineral solubility, K_{sp} corresponding to the ideal chemical formula of the mineral was used to calculate the dissolved Mn concentration in water (represented as $[Mn_{aq}]$). The K_{sp} of minerals are taken from Powell (1978), Ball & Nordstrom (1991), and Bruno (2014) [12-14].

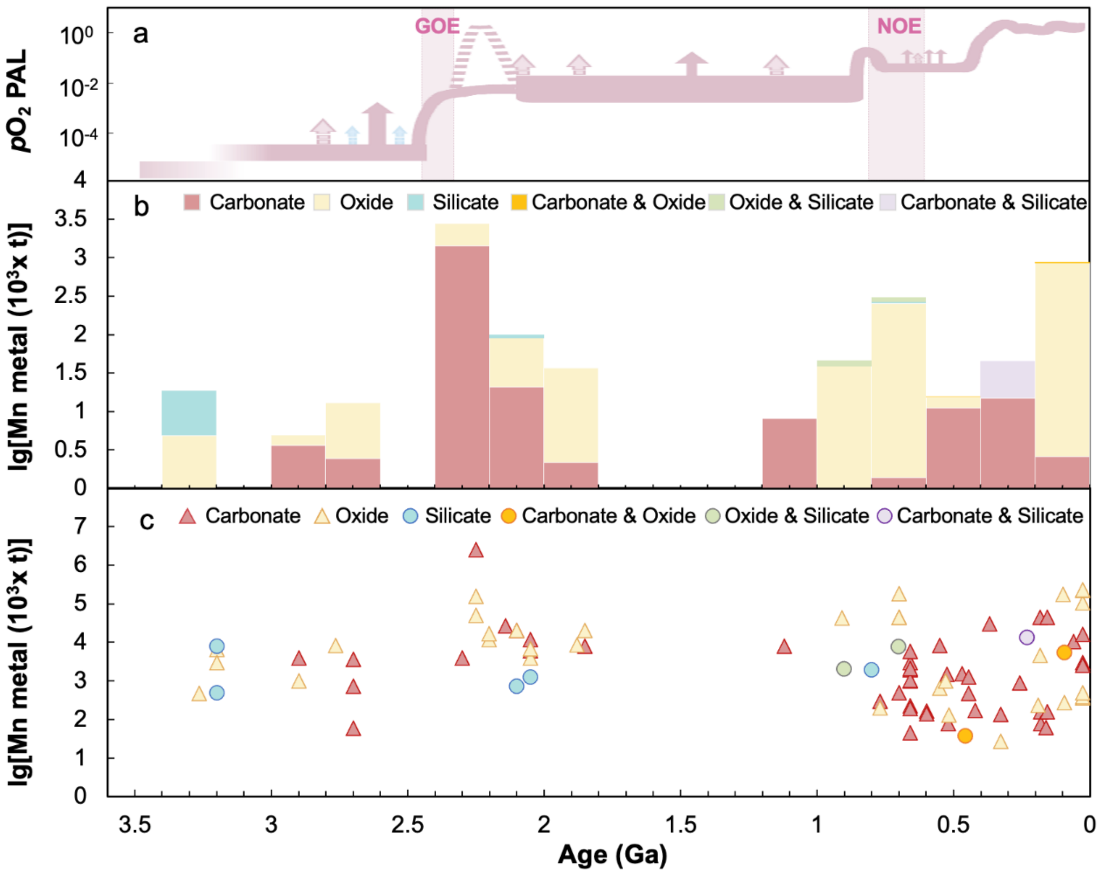

Figure S2. Temporal relationship between pO_2 and economic Mn deposits since 3.5 Ga. **a,** Current perspectives on pO_2 and the approximate timing of the GOE and the NOE (Lyons et al. [15]). **b,** Mass distribution of economic Mn deposits throughout geological history. **c,** Mass distribution of individual Mn deposits throughout 3.5 Gyr. Panels **b** and **c** are replotted from Maynard (2010) [16].

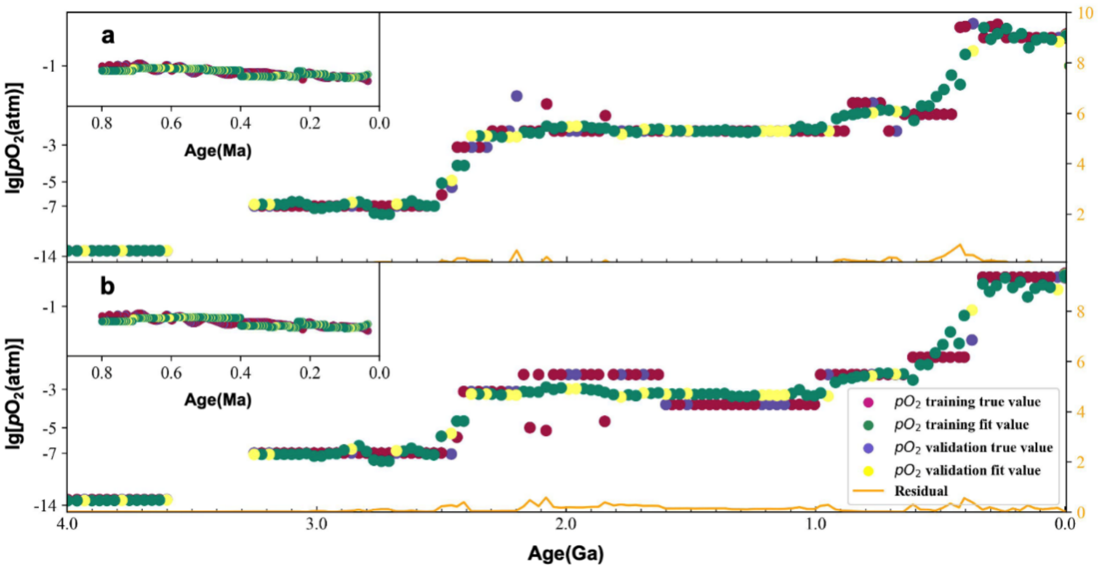

Figure S3. URD model validation. **a,** Deviation between network predictions and the maximum pO_2 range. **b,** Deviation from the minimum pO_2 range. Red scatters denote training data labels, purple scatters represent validation data, and green/yellow scatters show predictions from the trained network model. The difference between labels and predictions is depicted by an orange curve. Subgraphs (a) and (b) validate O_2 data from ice cores within 0~0.8 Ma. Alongside qualitative verification, quantitative error statistics of pO_2 predictions (MSE < 0.08, MAPE < 0.05, R^2 > 0.99) demonstrate the high accuracy of the URD model.

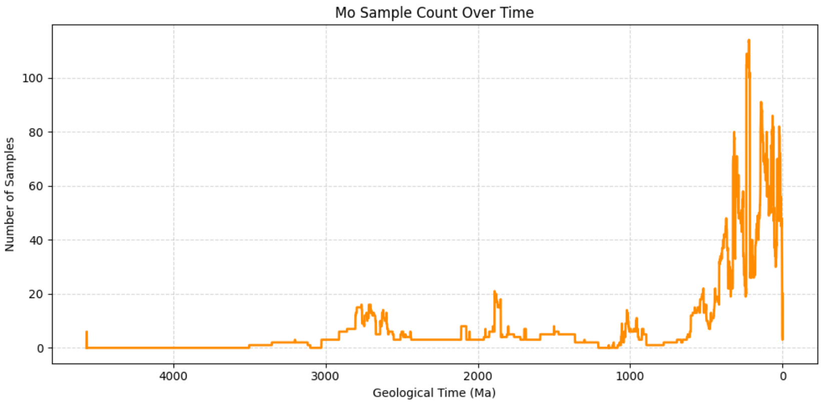

Figure S4. Deep-time evolution curve of Mo minerals. Note: maximum age was used to plot the curve, and only primary minerals are selected.

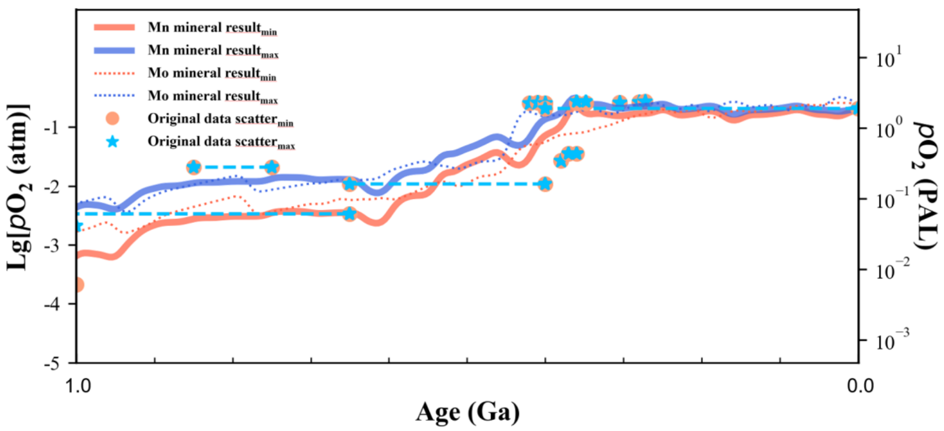

Figure S5. Comparison of pO_2 reconstructions based on Mn datasets and Mo datasets.

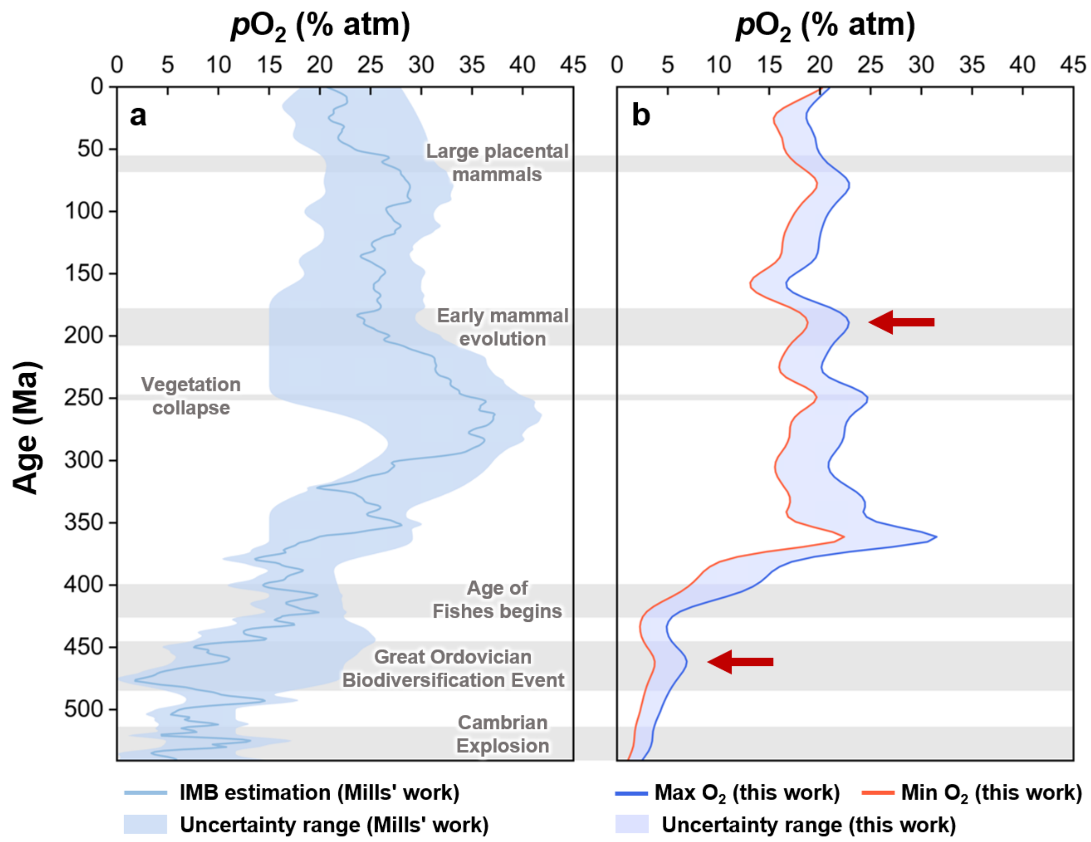

Figure S6. Comparison of reconstructed pO_2 throughout the Phanerozoic. (a) The phanerozoic pO_2 curve established by Mills [4]. The blue line represents pO_2 estimations using isotope mass balance (IMB) methods, and the blue shaded area represents the uncertainty range of their estimations. (b) The pO_2 curve reconstructed throughout the Phanerozoic in this work. The orange and blue curves depict the minimum and maximum reconstructed values, respectively. The horizontal gray areas denote selected key biotic events, encompassing both evolutionary radiations and crises, as detailed by Mills.

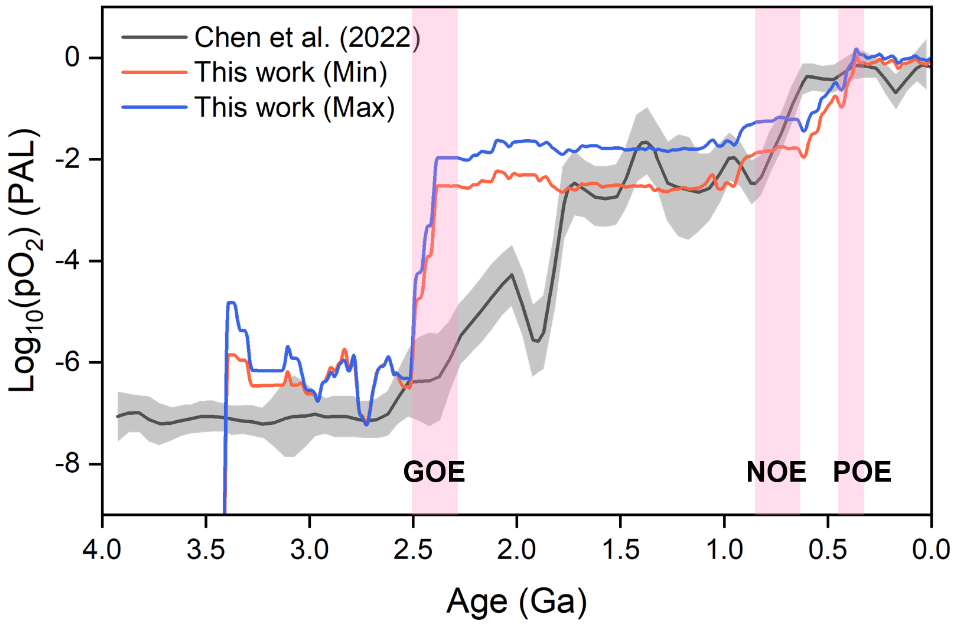

Figure S7. Comparison of reconstructed pO_2 with Chen et al. (2022) [9]. Black curve represents estimated atmospheric O_2 variation through time by Chen et al. (2022) [9]; the error bar (gray area) shows 2 standard deviation (2-SD) uncertainties. The orange and blue curves depict the minimum and maximum reconstructed atmospheric O_2 level in this work, respectively.

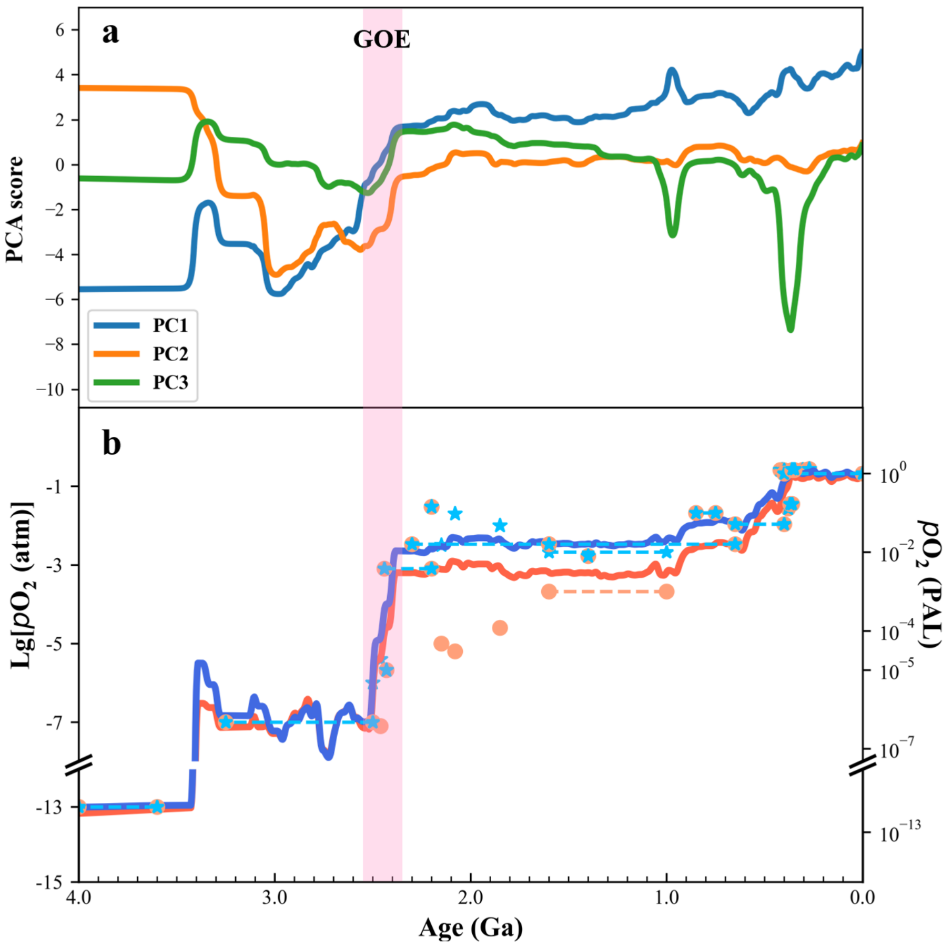

Figure S8. Principal component analysis of Mn mineral dataset (a) and the reconstructed pO_2 curve as shown in Fig. 3 (b). PC1, PC2, PC3 represent the top three principal components, which accounts for 46.8%, 15.9%, and 8.7% of the total variance, respectively. The principal component curves were smoothed using Kalman filtering methods.

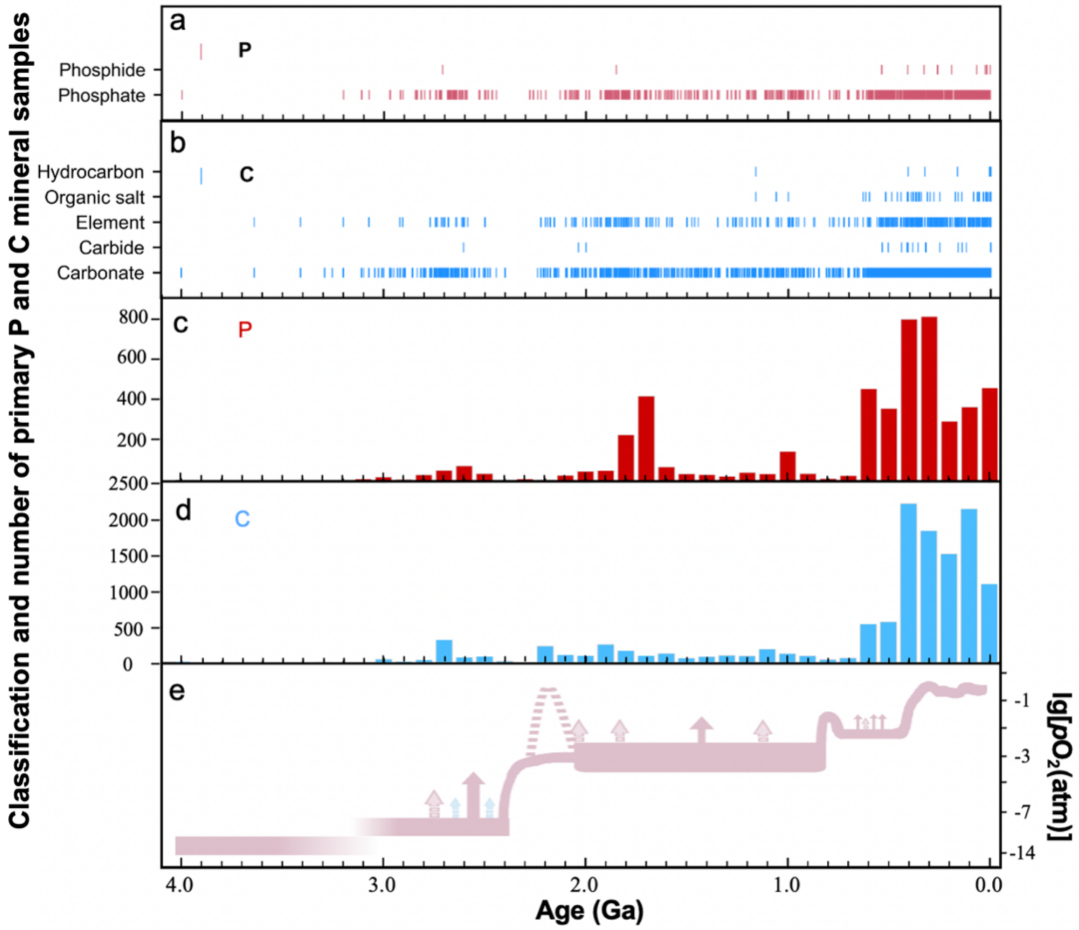

Figure S9. Co-evolution of primary P- and C-bearing minerals and pO_2 over 4.0 Gyr. a, Scatter diagram depicting the evolution of P-bearing minerals over 4.0 Gyr (classified by different chemical forms of P). **b,** Scatter diagram illustrating the evolution of C-bearing minerals over 4.0 Gyr (classified by different chemical forms of C). **c,** Box plot showing the statistics of P-bearing mineral counts over time. **d,** Box diagram plot showing the statistics of C-bearing mineral counts over time. **e,** Current perspectives on pO_2, sourced from Lyons et al. [15]. Each mineral count represents one occurrence of a sample-locality pair.

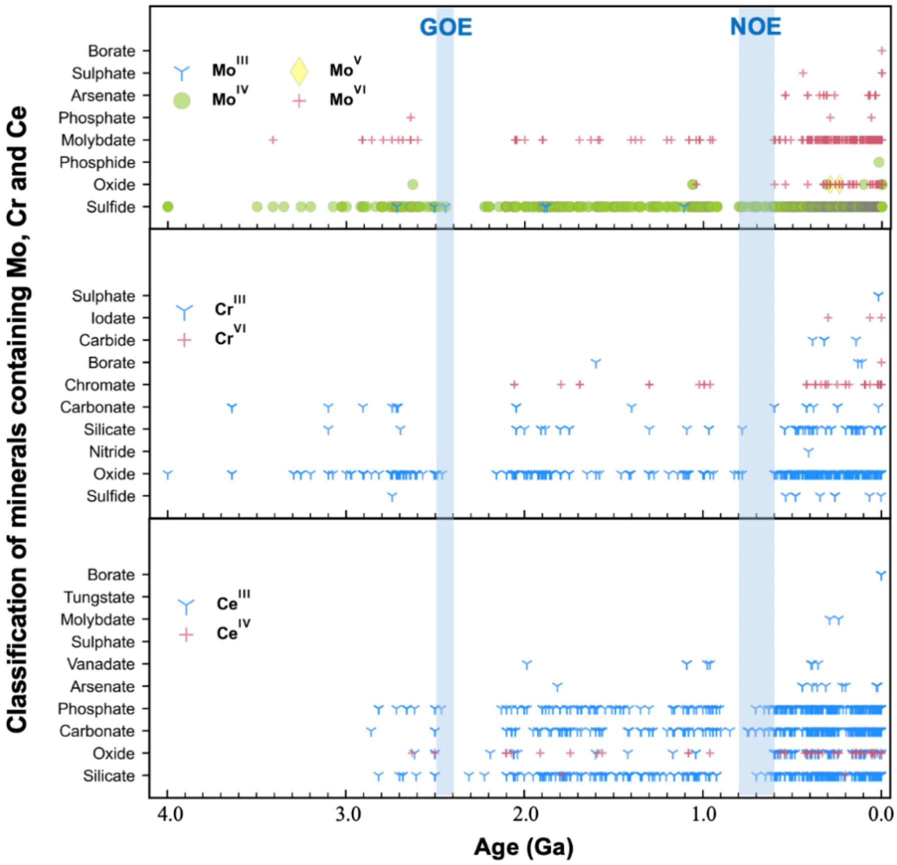

Figure S10. Co-evolution of Mo-, Cr- and Ce-bearing minerals with pO_2 over 4.0 Gyr. Individual minerals are distinguished by their various classifications and valence states. The two blue shadings represent the current perspective on the approximate timing of the GOE and the NOE (Lyons et al. [15]).

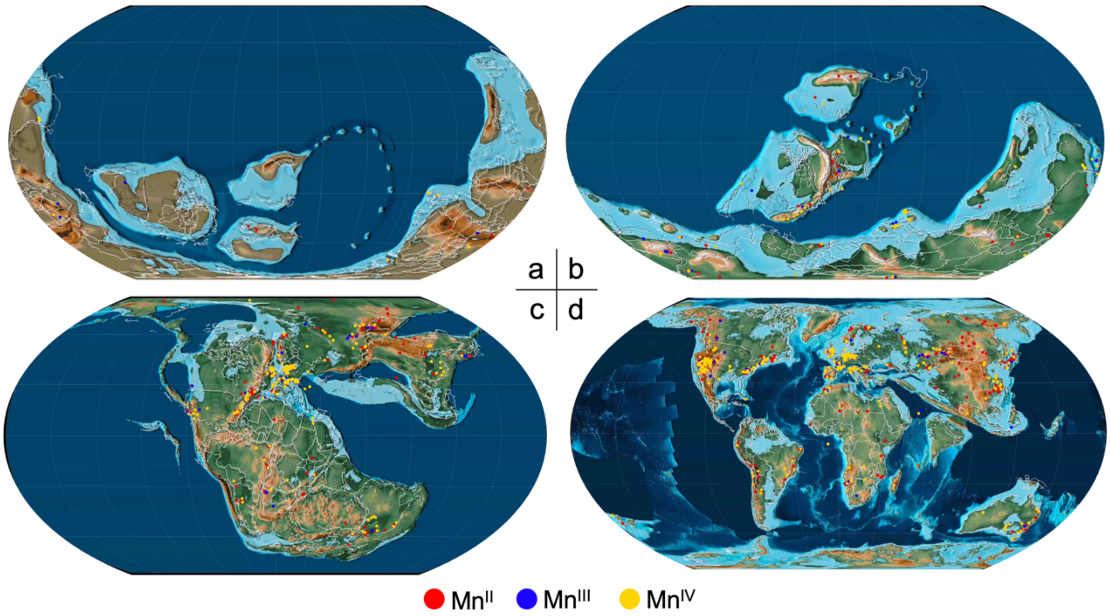

Figure S11. Distribution of Mn minerals over the past 545, 409, 205 and 50 Ma (corresponding to subfigures a-d). Subfigures b and c illustrate two supercontinent converges: Pannotia and Pangea, respectively.

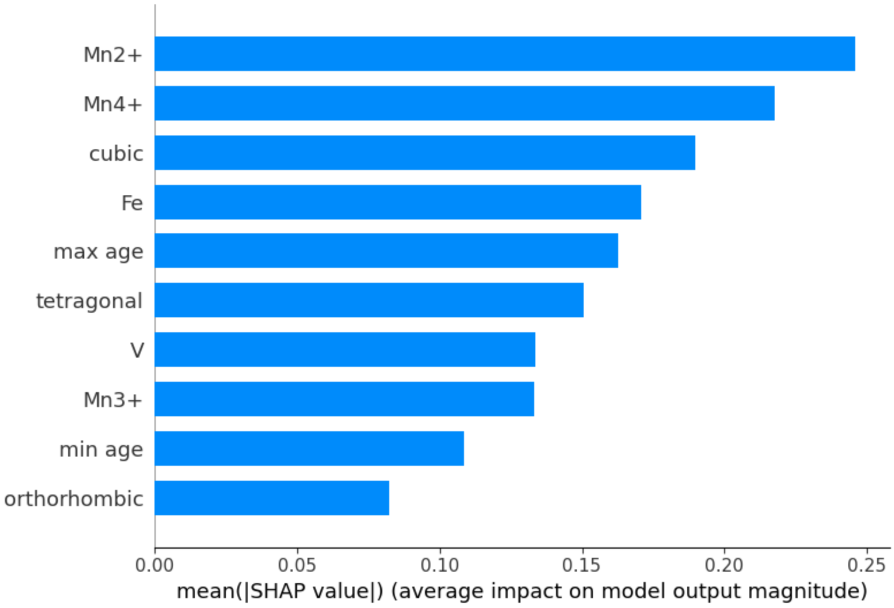

Figure S12. Ten key Mn mineral characteristics influencing maximum pO_2 near the NOE.

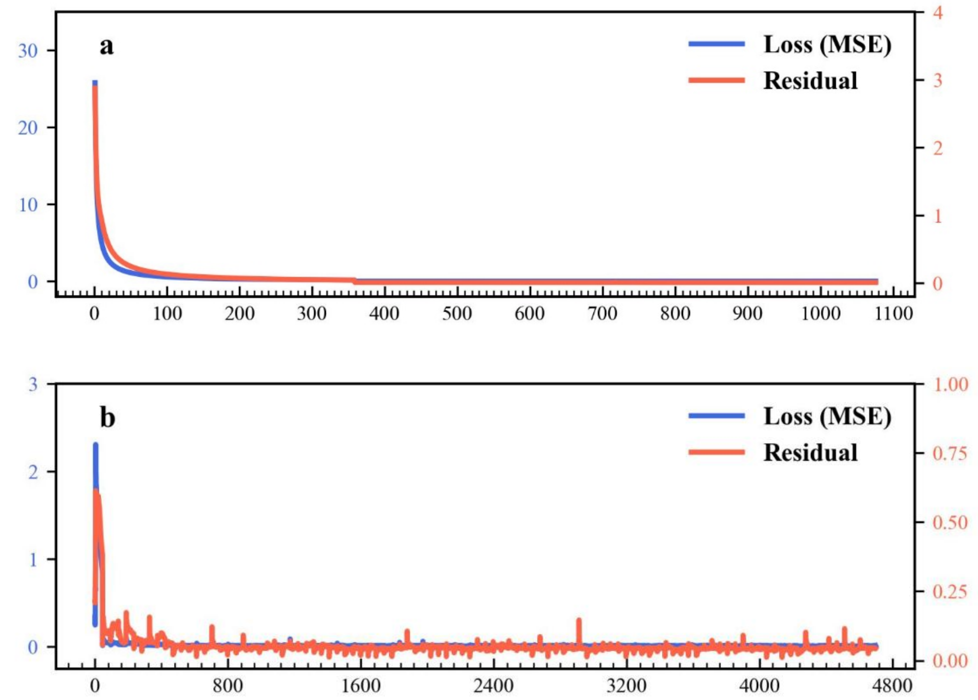

Figure S13. Network training curves. **a,** Loss values (MSE) indicated by the blue curve & residuals by the red curve. **b,** Logarithmic representations of the loss and residuals. The network demonstrates rapid convergence within 100 iterations.

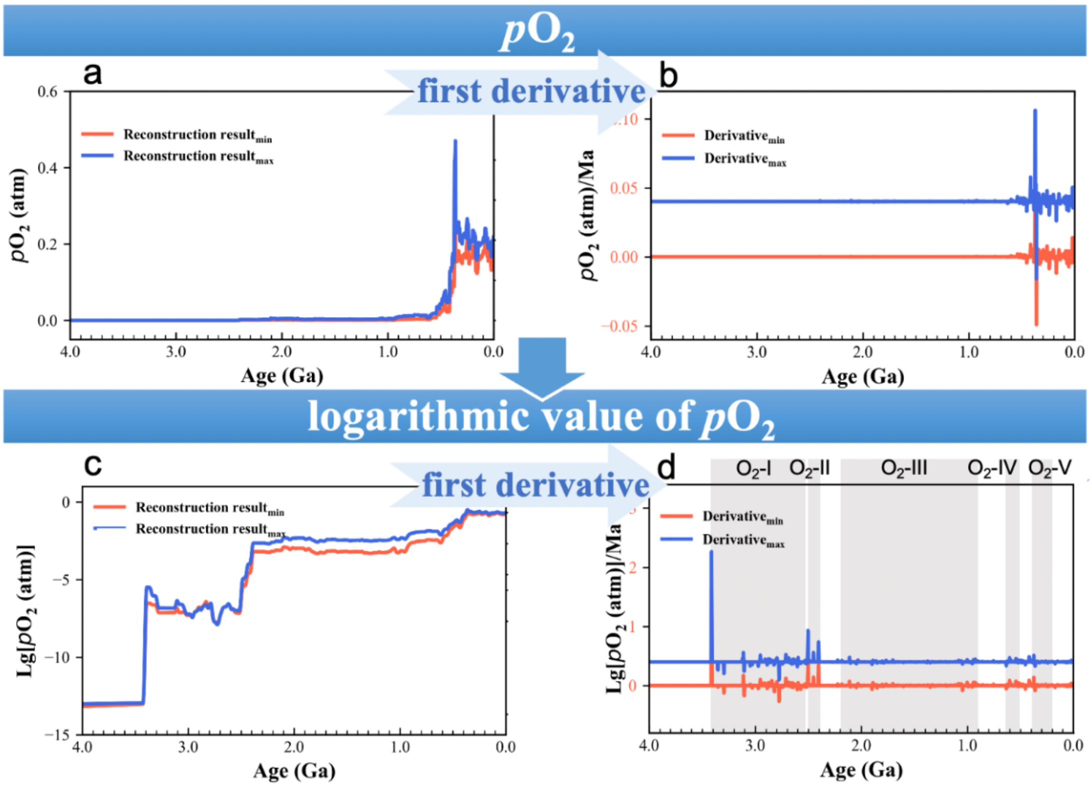

Figure S14. Illustration of mathematical transformation of pO_2.

Table S1. Descriptors used to numerically represent each Mn mineral.

Characters	Descriptors	Dimensions
Geologic age	Maximum/minimum age	2
Chemical composition	Maximum/minimum/average atomic number	3
Key elements	P, Fe, Cr, Ce, V, U, Mo	7
Mn valence states	Mn^{II}, Mn^{III}, Mn^{IV}	3
Structural symmetry	Monoclinic, Triclinic, Trigonal, Hexagonal, Orthorhombic, Tetragonal, Cubic, Amorphous, null	9
Chemical classifications	Sulfide, Silicate, Oxide, Carbonate, Tantalate, Niobate, Phosphate, Arsenate, Borate, Sulphate, Tungstate, Antimonate, Vanadate, Tellurite, Oxalate, Halide, Chromate	1

Table S2. pO_2 dataset based on geochemical proxies and models.

**Index**	**Age_{max} (Ga)**	**Age_{min} (Ga)**	**Upper/Lower limit**	**pO_2 (atm)[1]**
Atmosphere model[17]	4.00	3.60	/	1.0×10^{-13}
Paleosol[18]	2.46	2.46	/	7.9×10^{-8}~ 4.0×10^{-6}
Paleosol[18]	2.20	2.20	Lower limit	3.0×10^{-2}
Paleosol[18]	2.44	2.20	Upper limit	8.0×10^{-4}
Paleosol[18]	1.85	1.85	/	2.5×10^{-5}~ 1.0×10^{-2}
Paleosol[18]	2.08	2.08	/	6.3×10^{-6}~ 2.0×10^{-2}
Paleosol[18]	2.15	2.15	/	1.0×10^{-5}~ 3.2×10^{-3}
Weathering model-paleosol[19]	2.50	2.50	Upper limit	1.0×10^{-6}~ 1.0×10^{-7}
Preservation of detrital minerals[18]	3.25	2.50	Upper limit	1.0×10^{-7}
MIF-S[2, 20]	2.43	2.43	Lower limit	1.0×10^{-6}
Ce anomaly data (Ce/Ce*)[21]	1.60	1.00	/	2.1×10^{-4}~ 2.1×10^{-3}
Fe(II) oxidation kinetics[22]	0.85	0.75	/	2.1×10^{-2}
O-MIF[3, 23]	1.40	1.40	Lower limit	1.7×10^{-3}
Ce anomaly data (Ce/Ce*)[24]	2.30	1.60	/	3.4×10^{-3}
Ce anomaly data (Ce/Ce*)[24]	1.60	0.65	/	3.4×10^{-3}
Ce anomaly data (Ce/Ce*)[24]	0.65	0.40	/	1.1×10^{-2}
Ce anomaly data (Ce/Ce*)[24]	0.40	0.00	/	2.1×10^{-1}
GEOCARBSULF model[25]	0.42	0.42	Lower limit	2.6×10^{-1}
GEOCARBSULF model[25]	0.41	0.41	Lower limit	2.6×10^{-1}
GEOCARBSULF model[25]	0.40	0.40	Lower limit	2.6×10^{-1}
GEOCARBSULF model[25]	0.38	0.38	Lower limit	2.7×10^{-2}
GEOCARBSULF model[25]	0.37	0.36	/	3.6×10^{-2}
GEOCARBSULF model[25,26]	0.36	0.35	/	2.6×10^{-1}~ 2.8×10^{-1}

GEOCARBSULF model[25]	0.31	0.31	/	2.6×10^{-1}
GEOCARBSULF model[25]	0.28	0.28	/	2.7×10^{-1}
GEOCARBSULF model[25]	0.27	0.27	Lower limit	2.7×10^{-1}
Ice core record[27,28]	7.8×10^{-4}	0	/	2.0×10^{-1}~ 2.3×10^{-1}

Note: [1]The unit of pO_2 is standard atmospheric pressure (atm), 1 atm = 1.013×10^{-5} bar/Pa. [2]MIF-S: Sulfur mass-independent fractionation. [3]O-MIF: Mass-independent fractionation of oxygen isotopes.

Table S3. Four evolutionary stages of Mn minerals.

**Evolution stages**	**Age range (Ga)**	**Mineral species**	**Number of sample-locality pairs**
Stage M-I	4.00~3.29	8	8
Stage M-II	3.10~2.40	100	333
Stage M-III	2.20~0.91	228	1036
Stage M-IV	0.65~0.00	403	4359
After GOE	2.40~0.00	468	5543

Table S4. Quantitative evaluations in ten-fold cross-validation.

	**Amount of training data**	**[1]MSE [2]Max & [3]Min**	**[4]MAPE Max & Min**	**[5]R^2 Max & Min**
1	455	0.075 & 0.080	0.111 & 0.047	0.992 & 0.991
2	455	0.163 & 0.100	0.107 & 0.084	0.962 & 0.987
3	455	0.033 & 0.062	0.049 & 0.110	0.997 & 0.990
4	455	0.132 & 0.121	0.181 & 0.218	0.981 & 0.984
5	455	0.085 & 0.128	0.057 & 0.143	0.992 & 0.982
6	455	0.069 & 0.056	0.043 & 0.076	0.991 & 0.994
7	456	0.053 & 0.069	0.038 & 0.044	0.991 & 0.989
8	456	0.057 & 0.105	0.083 & 0.054	0.992 & 0.977
9	456	0.093 & 0.083	0.101 & 0.046	0.985 & 0.990
10	456	0.071 & 0.083	0.080 & 0.122	0.992 & 0.991
mean	/	0.083 & 0.089	0.085 & 0.094	0.988 & 0.988

Note: [1]MSE: Mean square error. [2]Max[O_2] & [3]Min[O_2]: Upper & Lower bounds of pO_2. [4]MAPE: Mean Absolute Percentage Error. [5]R^2: R-squared.

Table S5. Comparative experimental results.

Model	[1]MSE [2]Max & [3]Min	[4]MAPE Max & Min	[5]R^2 Max & Min
URD	0.071 & 0.073	0.042 & 0.045	0.990 & 0.991
Mean Feature	0.624 & 0.938	0.625 & 0.419	< 0.5 & < 0.5
Pad Zero	0.440 & 0.671	0.180 & 0.344	0.679 & < 0.5
Flag Identification	0.772 & 1.244	1.001 & 2.686	< 0.5 & < 0.5

Note: [1]MSE: Mean square error. [2]Max[O_2] & [3]Min[O_2]: Upper & Lower bounds of pO_2. [4]MAPE: Mean Absolute Percentage Error. [5]R^2: R-squared.

Table S6. Comparative evaluation metrics based on Mn and Mo datasets in 0−1.0 Ga.

Dataset	[1]MSE<br>[2]Max & [3]Min	[4]MAPE<br>Max & Min	[5]R^2<br>Max & Min
Mn mineral	0.048 & 0.051	0.030 & 0.036	0.942 & 0.951
Mo mineral	0.064 & 0.039	0.054 & 0.040	0.900 & 0.971

Note: [1]MSE: Mean square error. [2]Max[O_2] & [3]Min[O_2]: Upper & Lower bounds of pO_2. [4]MAPE: Mean Absolute Percentage Error. [5]R^2: R-squared.

Table S7. Predictions of pO_2 and their changing rates at each period by the URD model.

**Age (Ga)**	**Evolution period**	**Related events**	**pO_2 (atm)**	**Changing rate of lg[pO_2][1]**
3.4~2.5	Period I	Evolution of OP[2]	9.4×10^{-14}~3.0×10^{-7} (min)	1.1×10^{0} (min)
			1.1×10^{-13}~3.2×10^{-6} (max)	1.3×10^{-1} (max)
2.5~2.4	Period II	GOE	1.2×10^{-6}~6.3×10^{-4} (min)	2.5×10^{1} (min)
			4.0×10^{-6}~2.3×10^{-3} (max)	2.7×10^{1} (max)
2.2~0.9	Period III	Columbia ~ Rodinia	6.7×10^{-4}~2.4×10^{-3} (min)	6.5×10^{-3} (min)
			2.5×10^{-3}~1.0×10^{-2} (max)	1.6×10^{-2} (max)
0.65~0.50	Period IV	Panotia	3.4×10^{-3}~2.2×10^{-2} (min)	6.1×10^{0} (min)
			1.2×10^{-2}~4.1×10^{-2} (max)	4.0×10^{0} (max)
0.43~0.20	Period V	Pangea	2.4×10^{-2}~1.8×10^{-1} (min)	3.1×10^{0} (min)
			5.2×10^{-2}~2.2×10^{-1} (max)	1.4×10^{0} (max)

Note: [1]Changing rate of lg[pO_2] established by least-squares fitting. [2]OP, represents Oxygenic Photosynthesis.

Table S8. Mn minerals with the strongest correlation with pO_2 at each time period.

Mineral Phase	Chemical formula
**Period I (3.4~2.5 Ga)**	
**Mineral Phase**	**Chemical formula**
Pyrolusite	$Mn^{IV}O_2$
Hausmannite	$Mn^{II}Mn^{III}{}_2O_4$
Coronadite	$Pb(Mn^{IV}{}_6Mn^{III}{}_2)O_{16}$
**Period II (2.5~2.4 Ga)**	
**Mineral Phase**	**Chemical formula**
Vuorelainenite	$Mn^{II}V^{III}{}_2O_4$
Pyrolusite	$Mn^{IV}O_2$
Coronadite	$Pb(Mn^{IV}{}_6Mn^{III}{}_2)O_{16}$
Hausmannite	$Mn^{II}Mn^{III}{}_2O_4$
Ercitite	$NaMn^{III}(PO_4)(OH)\cdot 2H_2O$
**Period III (2.2~0.9 Ga)**	
**Mineral Phase**	**Chemical formula**
Vuorelainenite	$Mn^{II}V^{III}{}_2O_4$
Coronadite	$Pb(Mn^{IV}{}_6Mn^{III}{}_2)O_{16}$
Hollandite	$Ba(Mn^{IV}Mn^{III})_8O_{16}$
Pyrolusite	$Mn^{IV}O_2$
Pyrobelonite	$PbMn^{II}V^{V}O_4(OH)$
**Period IV (0.65~0.50 Ga)**	
**Mineral Phase**	**Chemical formula**
Cryptomelane	$K(Mn^{IV}{}_7Mn^{III})O_{16}$
Vuorelainenite	$Mn^{II}V^{III}{}_2O_4$
Lehnerite	$Mn^{II}(U^{VI}O_2)_2(PO_4)_2\cdot 8H_2O$
Momoiite	$Mn^{II}{}_3V^{III}{}_2(SiO_4)_3$
Coronadite	$Pb(Mn^{IV}{}_6Mn^{III}{}_2)O_{16}$
Hollandite	$Ba(Mn^{IV}Mn^{III})_8O_{16}$
Khristovite-(Ce)	$CaCe^{III}(MgAlMn^{II})[Si_2O_7][SiO_4]F(OH)$
**Period V (0.43~0.20 Ga)**	
**Mineral Phase**	**Chemical formula**
Zirsilite-(Ce)	$Na_{12}(Ce^{III},Na)_3Ca_6Mn^{II}{}_3Zr_3NbSi_{25}O_{73}(OH)_3(CO_3)\cdot H_2O$
Carbokentbrooksite	$Na_{12}(Na,Ce^{III})_3Ca_6Mn^{II}{}_3Zr_3NbSi_{25}O_{73}(OH)_3(CO_3)\cdot H_2O$
Santafeite	$(Ca,Sr,Na)_3(Mn^{II},Fe^{III})_2Mn^{IV}{}_2(V^{V}O_4)_4(OH,O)_5\cdot 2H_2O$
Vuorelainenite	$Mn^{II}V^{III}{}_2O_4$
Momoiite	$Mn^{II}{}_3V^{III}{}_2(SiO_4)_3$
Redcanyonite	$(NH_4)_2Mn^{II}[(U^{VI}O_2)_4O_4(SO_4)_2](H_2O)_4$
Lehnerite	$Mn^{II}(U^{VI}O_2)_2(PO_4)_2\cdot 8H_2O$
Coronadite	$Pb(Mn^{IV}{}_6Mn^{III}{}_2)O_{16}$

Fritzscheite	$Mn^{II}(UO_2)_2[(PO_4), (VO_4)]_2 \bullet 10H_2O$
Hydroxymanganopyro-chlore	$(Mn^{II},Th,Na,Ca,REE)_2(Nb,Ti)_2O_6(OH)$
Ikranite	$(Na,H_3O)_{15}(Ca,Mn^{II},REE)_6Fe^{III}{}_2Zr_3Si_{24}O_{66}(O,OH)_6Cl\cdot nH_2O$
Steenstrupine-(Ce)	$Na_{14}Ce^{III}{}_6Mn^{II}{}_2Fe^{III}{}_2Zr(PO_4)_7Si_{12}O_{36}(OH)_2\cdot 3H_2O$
Andrianovite	$Na_{12}(K,Sr,Ce^{III})_3Ca_6Mn^{II}{}_3Zr_3Nb(Si_{25}O_{73})(O,H_2O,OH)_5$
Manganonordite-(Ce)	$Na_3SrCeMn^{II}Si_6O_{17}$
Hollandite	$Ba(Mn^{IV}Mn^{III})_8O_{16}$

Supplementary Video (.mp4). Distribution of Mn minerals over the past 545 Ma. Red, blue and

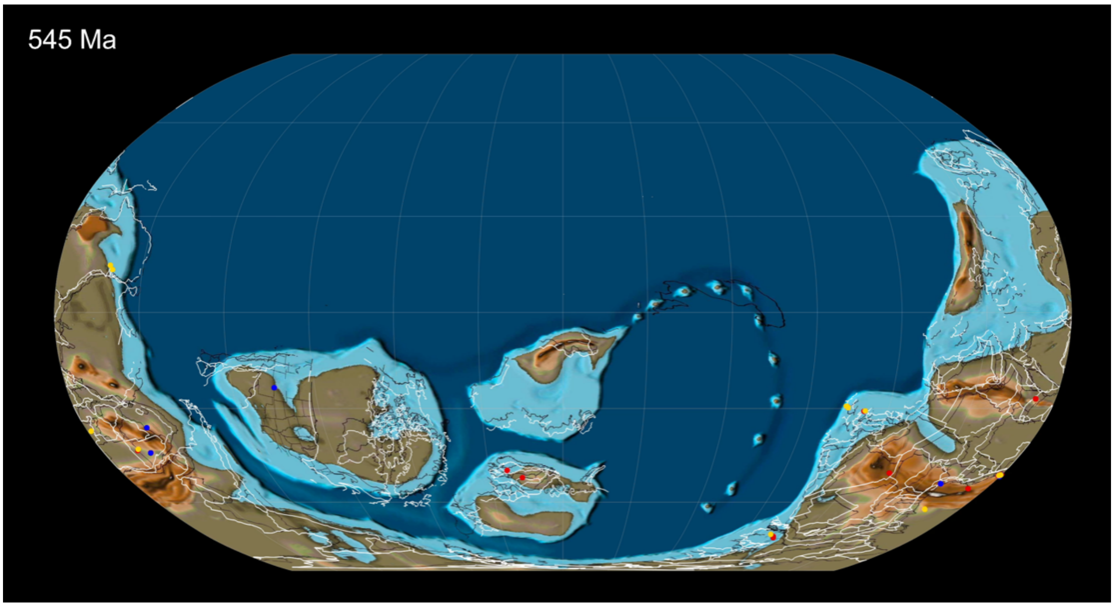

gold circles denote Mn^{II}, Mn^{III}, Mn^{IV} minerals, respectively, and the white curve represents plate boundaries (see Supplementary Video for complete details).

REFERENCES

1. Wang Z, Cai S, Chen G et al. Describe, explain, plan and select: interactive planning with llms enables open-world multi-task agents. Advances in Neural Information Processing Systems. 2023; 36: 34153-34189.
2. Swanson K, Wu E, Zhang A et al. From patterns to patients: Advances in clinical machine learning for cancer diagnosis, prognosis, and treatment. Cell. 2023; 186(8): 1772-1791.
3. Mehrish A, Majumder N, Bharadwaj R et al. A review of deep learning techniques for speech processing. Information Fusion. 2023; 99: 101869.
4. Mills BJ, Krause AJ, Jarvis I et al. Evolution of atmospheric O2 through the Phanerozoic, revisited. Annual Review of Earth and Planetary Sciences. 2023; 51: 253-276.
5. Edwards CT, Saltzman MR, Royer DL et al. Oxygenation as a driver of the Great Ordovician Biodiversification Event. Nature Geoscience. 2017; 10(12): 925-929.
6. Falkowski PG, Katz ME, Milligan AJ et al. The rise of oxygen over the past 205 million years and the evolution of large placental mammals. Science. 2005; 309(5744): 2202-2204.
7. Newby SM, Owens JD, Schoepfer SD et al. Transient ocean oxygenation at end-Permian mass extinction onset shown by thallium isotopes. Nature Geoscience. 2021; 14(9): 678-683.
8. Stockey RG, Cole DB, Farrell UC et al. Sustained increases in atmospheric oxygen and marine productivity in the Neoproterozoic and Palaeozoic eras. Nature Geoscience. 2024; 17(7): 667-674.
9. Chen G, Cheng Q, Lyons TW et al. Reconstructing Earth’s atmospheric oxygenation history using machine learning. Nature Communications. 2022; 13(1): 5862.
10. Chen X, Ostrander CM, Holdaway BJ et al. Transient marine bottom water oxygenation on continental shelves by 2.65 billion years ago. Nature Geoscience. 2025: 1-7.
11. Liang X, Stüeken EE, Alessi DS et al. A seawater oxygen oscillation recorded by iron formations prior to the Great Oxidation Event. Nature Geoscience. 2025: 1-6.
12. Powell R. The thermodynamics of pyroxene geotherms. Philosophical Transactions of the Royal Society of London Series A, Mathematical and Physical Sciences. 1978; 288(1355): 457-469.
13. Ball JW, Nordstrom DK. User’s manual for WATEQ4F, with revised thermodynamic data base and text cases for calculating speciation of major, trace, and redox elements in natural waters. US Geological Survey; 1991 (DHHS publication no.: Report Number)| (GPO o. Document Number)|.
14. Bruno DRL-TJ. CRC Handbook of Chemistry and Physics, CRC Press, 2014: CRC Handbook of Chemistry and Physics: Bukupedia, 2014.
15. Lyons TW, Diamond CW, Planavsky NJ et al. Oxygenation, life, and the planetary system during Earth's middle history: An overview. Astrobiology. 2021; 21(8): 906-923.
16. Maynard JB. The chemistry of manganese ores through time: a signal of increasing diversity of earth-surface environments. Economic Geology. 2010; 105(3): 535-552.
17. Catling DC, Glein CR, Zahnle KJ et al. Why O_2 is required by complex life on habitable planets and the concept of planetary “Oxygenation Time”. Astrobiology. 2005; 5(3): 415-438.
18. Kanzaki Y, Murakami T. Estimates of atmospheric O_2 in the Paleoproterozoic from paleosols. Geochimica et Cosmochimica Acta. 2016; 174: 263-290.
19. Murakami T, Sreenivas B, Sharma SD et al. Quantification of atmospheric oxygen levels during the Paleoproterozoic using paleosol compositions and iron oxidation kinetics.

Geochimica et cosmochimica acta. 2011; 75(14): 3982-4004.
20. Claire MW, Kasting JF, Domagal-Goldman SD et al. Modeling the signature of sulfur mass-independent fractionation produced in the Archean atmosphere. Geochimica et Cosmochimica Acta. 2014; 141: 365-380.
21. Bellefroid EJ, Hood AvS, Hoffman PF et al. Constraints on Paleoproterozoic atmospheric oxygen levels. Proceedings of the National Academy of Sciences. 2018; 115(32): 8104-8109.
22. Wang C, Lechte MA, Reinhard CT et al. Strong evidence for a weakly oxygenated ocean–atmosphere system during the Proterozoic. Proceedings of the National Academy of Sciences. 2022; 119(6): e2116101119.
23. Liu P, Liu J, Ji A et al. Triple oxygen isotope constraints on atmospheric O_2 and biological productivity during the mid-Proterozoic. Proceedings of the National Academy of Sciences. 2021; 118(51): e2105074118.
24. Liu X-M, Kah LC, Knoll AH et al. A persistently low level of atmospheric oxygen in Earth's middle age. Nature Communications. 2021; 12(1): 351.
25. Berner RA. GEOCARBSULF: a combined model for Phanerozoic atmospheric O_2 and CO_2. Geochimica et Cosmochimica Acta. 2006; 70(23): 5653-5664.
26. Berner RA. Phanerozoic atmospheric oxygen: New results using the GEOCARBSULF model. American Journal of Science. 2009; 309(7): 603-606.
27. Stolper D, Bender M, Dreyfus G et al. A Pleistocene ice core record of atmospheric O_2 concentrations. Science. 2016; 353(6306): 1427-1430.
28. Yan Y, Brook EJ, Kurbatov AV et al. Ice core evidence for atmospheric oxygen decline since the Mid-Pleistocene transition. Science advances. 2021; 7(51): eabj9341.
